# A Four-Year Field Program Investigating Long-Term Effects of Repeated Exposure of Honey Bee Colonies to Flowering Crops Treated with Thiamethoxam

**DOI:** 10.1371/journal.pone.0077193

**Published:** 2013-10-23

**Authors:** Edward Pilling, Peter Campbell, Mike Coulson, Natalie Ruddle, Ingo Tornier

**Affiliations:** 1 JSC International Limited, Harrogate, North Yorkshire, United Kingdom; 2 Syngenta Limited, Jealott’s Hill Research Station, Bracknell, Berkshire, United Kingdom; 3 Eurofins Agroscience Services, EcoChem GmbH, Niefern-Öschelbronn, Germany; University of Maryland, United States of America

## Abstract

Neonicotinoid residues in nectar and pollen from crop plants have been implicated as one of the potential factors causing the declines of honey bee populations. Median residues of thiamethoxam in pollen collected from honey bees after foraging on flowering seed treated maize were found to be between 1 and 7 µg/kg, median residues of the metabolite CGA322704 (clothianidin) in the pollen were between 1 and 4 µg/kg. In oilseed rape, median residues of thiamethoxam found in pollen collected from bees were between <1 and 3.5 µg/kg and in nectar from foraging bees were between 0.65 and 2.4 µg/kg. Median residues of CGA322704 in pollen and nectar in the oilseed rape trials were all below the limit of quantification (1 µg/kg). Residues in the hive were even lower in both the maize and oilseed rape trials, being at or below the level of detection of 1 µg/kg for bee bread in the hive and at or below the level of detection of 0.5 µg/kg for hive nectar, honey and royal jelly samples. The long-term risk to honey bee colonies in the field was also investigated, including the sensitive overwintering stage, from four years consecutive single treatment crop exposures to flowering maize and oilseed rape grown from thiamethoxam treated seeds at rates recommended for insect control. Throughout the study, mortality, foraging behavior, colony strength, colony weight, brood development and food storage levels were similar between treatment and control colonies. Detailed examination of brood development throughout the year demonstrated that colonies exposed to the treated crop were able to successfully overwinter and had a similar health status to the control colonies in the following spring. We conclude that these data demonstrate there is a low risk to honey bees from systemic residues in nectar and pollen following the use of thiamethoxam as a seed treatment on oilseed rape and maize.

## Introduction

Honey bees provide a vitally important role, both ecologically by pollinating wild plants, and economically by providing a pollination service to a variety of crops around the world [1]. Numerous studies have, however, reported a decline in honey bee health and numbers of colonies in recent years [2], [3]. Bee keepers in many countries have reported a decline in the ability of colonies to successfully survive the winter, while others report the sudden disappearance of all but a few bees, with just the young and the queen remaining [4], [5]. Many factors may have contributed to this decline in health, for example the spread of parasites and pathogens [6], reduction in available forage [7], beekeeping management practices (for example *Varroa destructor* control and the development of resistance to treatments), movement of colonies, weather and climate change [8]. Exposure to certain pesticides is also another factor that has been implicated in bee health decline [9]. In particular, the use of neonicotinoid insecticides in crops where bees forage has been reported as a potentially contributing factor [10], [11].

To develop our understanding of potential risk to honey bees from systemic residues in pollen and nectar associated with neonicotinoid seed treated crops, it is important to conduct in-use field trials where colonies exposed to seed treated crops are monitored throughout the bee season and the sensitive overwintering period. Whilst there are a number of field studies in the literature which have concluded no significant risk to honey bee colonies from pollen and nectar residues associated with neonicotinoid seed treated crops [12], [13], few have investigated effects on the overwintering success or covered consecutive exposures over multiple years.

The objectives of this investigation were two-fold;

- Quantify the level of honey bee exposure to residues of thiamethoxam and its primary metabolite CGA322704 (clothianidin, IUPAC name N-[(2-chloro-5-thiazoyl)methyl]-N’-methyl-N’’-nitroguanidine) in pollen and nectar collected from maize and oilseed rape grown from treated seed and determine subsequent exposure and persistence of residues brought back to the colony and presence in bee products.
- Investigate if exposure to such residues in pollen and nectar from field treated maize and oilseed rape have a detrimental effect on colony strength and survival following repeated single treatment crop exposure each year over a four year period.

This paper therefore reports on two studies, the first study quantifies the residues of thiamethoxam and CGA322704 in pollen and nectar collected by foraging bees. The study included an investigation of potential carryover of residues from rotation of seed treated barley to a following crop of seed treated oilseed rape, and from a two year seed treated maize rotation. In addition, this study quantified residues in the hive by analysing bee bread stored in cells, nectar, honey, wax and royal jelly. The study was conducted under field conditions that maximized potential exposure to pollen and nectar residues by maintaining bees in a tunnel built over the thiamethoxam treated crop to prevent them from foraging elsewhere. The second study investigated the long-term risk to honey bee colonies, including the sensitive overwintering period, from four years of consecutive single treatment crop exposures to flowering maize and oilseed rape grown from thiamethoxam treated seeds at maximum label rates recommended for insect control in Europe. This study was conducted for regulatory purposes to specifically investigate the effects of thiamethoxam and therefore was conducted in such a way as to avoid any confounding effects of exposure to other pesticide applications.

## Materials and Methods

### Ethics Statement

All necessary permits were obtained for the described field studies, permission was granted at each location by the owner of the farm land used and the field studies did not involve endangered or protected species.

### Residues in Pollen, Nectar and Bee Products Study

A total of 12 residue trials were conducted in the Alsace, Picardie, Champagne and Midi-Pyrénées regions of France. These included three oil seed rape trials drilled in 2005 and three maize trials drilled in 2005. In addition, to investigate any potential carry-over of residues that may occur following typical crop rotation practices, three further maize trials were drilled on the same fields in 2006 and three trials were conducted with seed treated spring barley followed by seed treated oilseed rape. In the maize trials, seed was treated with a flowable concentrate mixture of thiamethoxam (420 g/L) and two fungicides according to normal seed treating practice, metalaxyl-M (1.33 g/L) and fludioxinil (3.33 g/L). In the oilseed rape trials, rape seed (variety Roxet) was treated with a flowable concentrate mixture of thiamethoxam (280 g/L), metalaxyl-M (33.3 g/L) and fludioxinil (8.0 g/L). All trials used the maximum approved label rate for thiamethoxam as a seed treatment; in maize, the nominal rate was 88.2 g a.s./ha (0.85 mg a.s./seed); in oilseed rape, the rate was 12.6 g a.s./ha (0.02 mg a.s./seed); and in spring barley, the rate was 77 g a.s./ha (0.03 mg a.s./seed). At each trial site, before the start of flowering, mesh covered tunnels were set up enclosing sections of the crop. Honey bees were then exposed to the crop inside three replicate tunnels constructed over the treated crop and one tunnel over an adjacent control field. Each tunnel was 40 m long × 5 m wide and contained a bee colony with 1 queen and approximately 10,000 to 20,000 bees in two boxes with 10 combs each. At the start of flowering (BBCH 63 for maize, BBCH 60–62 for oilseed rape) a hive was placed in each tunnel. The hives remained in the tunnel for the entire duration of the exposure period (between 4 and 10 days in the maize trials and between 9 and 14 days in the oilseed rape trials).

Whole plants were collected on 3 sampling days during the flowering period; 1, 7 and 9 days after exposure of the hives in the treated tunnels and on day 1 in the control tunnel. The plants were cut off above the roots and stored deep frozen (<−18°C) before analysis.

Forager bees were collected on 3 sampling days during the exposure period to the flowering crop; 1, 7 and 9 days after exposure to the crop. Hive entrances were sealed before the sampling and the forager bees were subsequently collected as they returned to the hive by brushing them into a box filled with dry ice. The bees were stored deep frozen (<−18°C) until preparation of the honey stomachs and pollen loads. The preparation of the bee samples was conducted at LAVES bee institute in Celle, Germany. Pollen loads of single bees were removed and pooled from the same sample then transferred back to the freezer for storage. To prepare the honey stomachs, bees were fixed at their thorax and their abdomens were stretched flat with tweezers. The abdomen or tergit plates of each bee were removed, so that the honey stomach was freed and could be held at the lowest part of the oesophagus. The honey stomach was removed and the nectar collected into a vial which was subsequently stored in a freezer.

Bee bread stored in hive cells, nectar and wax samples from the combs were collected once on the day hives were set up, at 1, 7, 9 and 20 days after exposure of the bees to the crop and thereafter at monthly intervals until the end of September. Where possible, honey was collected from the colonies in the same way as the pollen, nectar and wax. Honey samples were only taken from capped cells to ensure that the water content of the nectar was <20% and thereby had changed into honey.

Bee product samples were extracted by vigorous shaking with methanol:0.2% formic acid in ultra-pure water (50∶50 v/v). Sample clean-up was performed by solid-phase extraction (SPE) using Oasis HLB cartridges. Plant samples were extracted with methanol:water (50∶50 v/v); an aliquot was taken and diluted with ultra-pure water.

The samples of bee and hive products were analysed for residues of thiamethoxam and its metabolite CGA322704. The residue analytical methods employed were fully validated according to SANCO/825/00. During the residue sample analysis phase, analytical grade standards (>95% purity) were used for all analytical determinations, and procedural recoveries were included alongside each analytical batch. Acceptable detector linearity was established to ensure that the quantitative analysis was conducted within the working range of the detector. Analysis was conducted by high performance liquid chromatography with triple quadrupole mass spectrophotometric detection (LC-MS/MS) using matrix matched standards. In bee nectar, hive wax, hive nectar and honey the limit of quantification (LOQ) of the method was 0.5 and 1 µg/kg for thiamethoxam and CGA322704, respectively. The LOQ of the method was 1 µg/kg for both thiamethoxam and CGA322704 in bee pollen, bee bread stored in cells and whole plant samples. The results of the residue analysis are presented as box and whisker plots, showing the median values with upper and lower quartile values and the full range of the data to show the variation. In the vast majority of cases it is not possible to derive a mean nor a standard error or standard deviation for the dataset because of the high proportion of values that are below the LOQ, so none are given.

### Multiple Exposure Study

Three long-term overwintering trials were established in maize in the Lorraine, Alsace and Aveyron regions of France in 2006, and two trials in oilseed rape in the Picardie and Alsace regions in 2005. Each trial site was isolated from other bee attractive crops or other maize and oilseed rape fields to avoid diluting exposure to potential residues of thiamethoxam. At each trial site, there was one control field and one treated field separated by approximately 2 km minimizing the movement of bees between fields. Each field was approximately 2 ha, thus providing adequate forage for the colonies and minimizing the incentive for bees to forage elsewhere. All trials used the maximum approved label rate for thiamethoxam as a seed treatment, in maize the nominal rate was 88.2 g a.s./ha (0.85 mg a.s./seed) and in oilseed rape the rate was 12.6 g a.s./ha (0.02 mg a.s./seed). At each site, six colonies were placed adjacent to the control field and six colonies adjacent to the treated field during the entire flowering period of the crop (exposure phase). The colonies were queen right at the start of the study and normalised as far as practically possible based on age-structure and extent of brood. Colonies were housed in two brood bodies comprising a brood chamber and a standard amount of stores with 5–8 brood combs plus 15–20 food combs.

**Figure 1:**
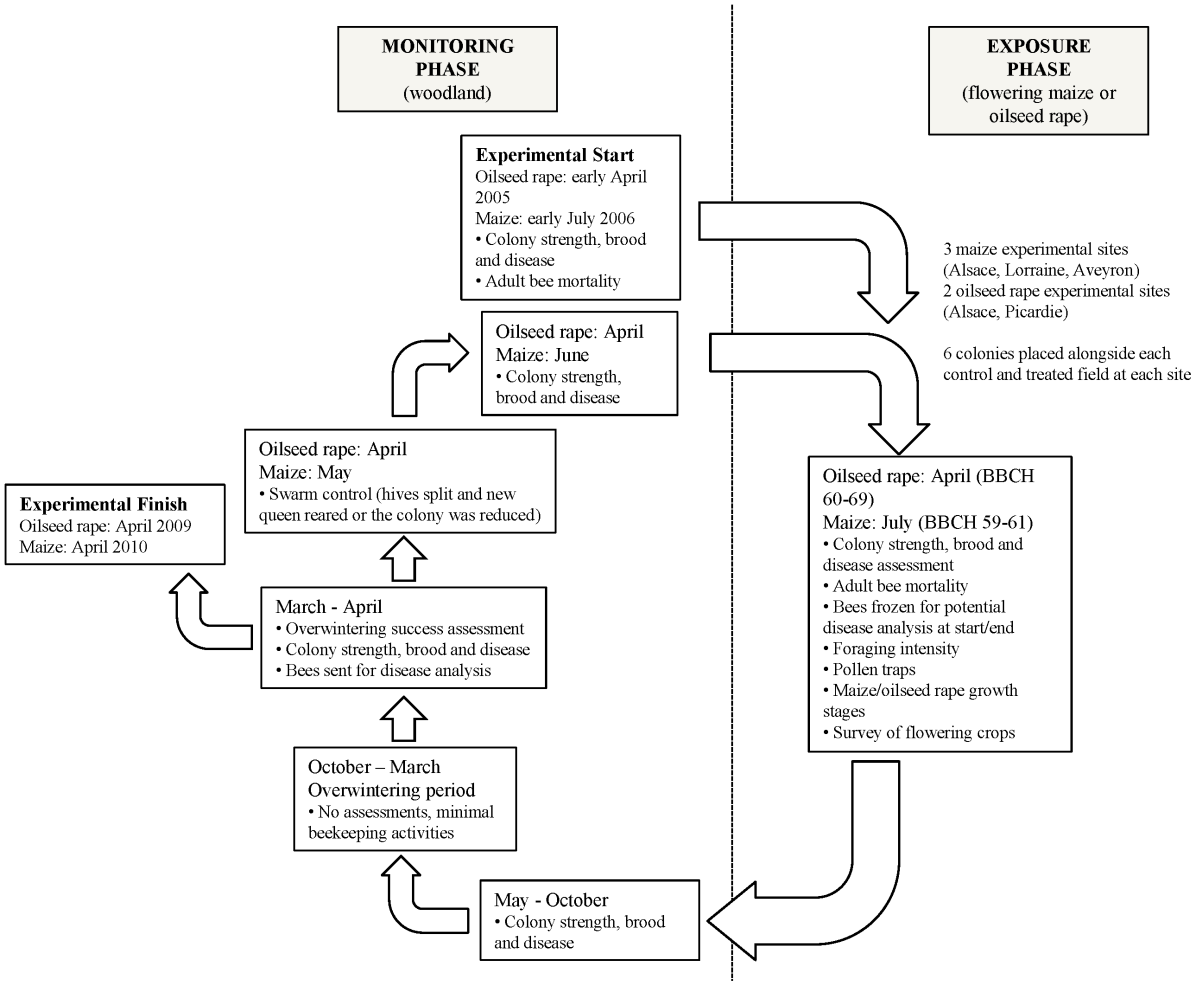
An overview of the 4 year multiple exposure field trial design in maize and oilseed rape.

After flowering, as this was a regulatory study assessing the specific effects of thiamethoxam, it was necessary to maintain the colonies away from further pesticide exposures to avoid potential ambiguity of any effects noted. Therefore the colonies were maintained at a woodland site, without extensive agricultural crops attractive to bees, in the Alsace region of France for the northern trials and in the region of Midi-Pyrénéés for the southern trial. Here the colony and brood development was monitored throughout the year and, in particular, before and after the overwintering period. At these monitoring sites the control and treated colonies were kept 2 km apart to avoid any cross-contamination of residues. An overview of the trial process is given in Figure 1.

The number of dead adult bees in front of the hives was assessed using dead bee traps and linen sheets which were emptied every day throughout the exposure period while the crop was in flower. The number of dead bees was also recorded using a linen sheet in three distinct places within the field plot to assess if bees were dying whilst foraging. The dead bee assessments started each year while the colonies were in the monitoring location (*i.e.* prior to exposure in the flowering crop); mortality was assessed in dead bee traps every day over a period of 5 days before they were due to be moved to the exposure sites. The colonies were relocated to the treated and control fields for the exposure phase of the trial at the start of flowering. In the maize trial, the dead bee assessments in the treated and control fields started when the crop reached BBCH 51–63 (*i.e*. the start of flowering) and then continued once a day until most of the plants (>80%) had reached BBCH 67–69. In the Alsace the duration of the exposure period in maize was 5, 8, 6 and 23 days in 2006, 2007, 2008 and 2009, respectively. In Lorraine, the exposure was for 5, 8, 7 and 19 days in 2006, 2007, 2008 and 2009, respectively and in Aveyron exposure was for 6, 6, 6, and 23 days in 2006, 2007, 2008 and 2009, respectively. The exposure period in 2009 was extended in each trial by planting two different varieties of maize (AADHOC and NK Perform) at two different times to extend the flowering and exposure period. In oilseed rape, the dead bee assessments were conducted every day for 9 days starting at BBCH 60–62 and subsequently every 2–3 days up to BBCH 69. In the Alsace the duration of the exposure period in oilseed rape was 19, 19, 13 and 22 days in 2005, 2006, 2007 and 2008, respectively. In Picardie the exposure period was 21, 17, 12 and 21 days in 2005, 2006, 2007 and 2008, respectively.

Flight activity at the hive entrance was assessed every day during the exposure period by counting the number of bees entering and leaving the hive over a one-minute period. Behavior observations and any potential sub-lethal effects were also recorded, for example hyperactivity, excessive leg-grooming or fanning at the hive entrance. Foraging activity in the field plots was assessed in five designated areas (each 5 m×5 m) regularly distributed over the field. The number of bees actively foraging or flying around the anthers of flowering maize or oilseed rape flowers within one minute was recorded twice a day during the main foraging activity period on 0–9 days after set-up (DAS) of the hives in the flowering crop and then twice a day every 2–3 days up until approximately day 19 DAS. All colonies were equipped with pollen traps during the time of exposure to allow determination of the pollen source at each test field. Pollen from the traps was collected three times during the exposure period in each crop and was subsequently frozen before taxonomic identification of pollen types.

The following assessments were made on the condition of the colonies and development of their brood before and after the exposure phase of the trial:

- Strength of the colony (estimation of adult worker bee numbers based on the Liebefeld method [14]
- Presence of a healthy egg-laying queen and freshly laid (one day old) eggs
- Estimate of the pollen storage area and area with nectar (in % of the comb area)
- Estimate of the area containing eggs, larvae and capped cells (in % of the comb area)
- Weight of the colony

Each hive was placed on a specific hive-scale for the duration of the study (except during the overwintering periods). The weight of the complete hives (including frames of comb and bees) was automatically recorded at two-hourly intervals to determine the daily maximum weight of each colony. Therefore, if the colonies exposed to treated crops deteriorated and stores declined, or if substantial numbers of foraging bees were not able to return to the hive following exposure, the weight of the hives would be reduced compared to the control.

To restrict the urge for the colonies to swarm, in 2006 and 2007 in the oilseed rape trials and in 2007 and 2008 in the maize trials, colonies were split in late spring before exposure to the treated crop, into a nucleus colony and the original colony. The old queen and a proportion of adult worker bees were transferred to a nucleus hive with 1–2 combs of brood and 1–2 combs of stores from the original hive. The majority of bees, with brood and stores remained in the original colony and were encouraged to raise a new healthy, egg-laying queen. Only the original colonies were then used for the next exposure phase of the field trial. In 2008 in the oilseed rape trials and in 2009 in the maize trials, rather than splitting the colonies, the colony size was reduced by simply removing some brood and store combs. The empty space in the hives was then filled with empty combs and the old queen left in the original colony. In March or April of each year, a brood assessment was conducted to determine the overwintering success of each colony. These colonies were then taken forward to the subsequent exposure phase in the maize and oilseed rape field plots.

A detailed assessment of brood development was also conducted before and after the exposure period. At the end of flowering, colonies were relocated to the monitoring site where the colony health and strength as well as brood development were assessed every 10 days.

To estimate the level of exposure of honey bees to residues of thiamethoxam and its metabolite CGA322704, samples of whole plant material and pollen (and nectar from oilseed rape) from the plants were taken from both the treated and control plots for residue analysis. Samples of forager bees were taken for residue and taxonomic analysis of pollen loads (and residue analysis in honey stomachs for the oilseed rape trials). Approximately 50 forager bees from each of the six colonies per treatment per trial were collected as they returned to the hive by a hand-held vacuum, which sucked the bees into a container filled with dry ice. Pollen and nectar samples were extracted and analysed as previously described in the specific residues study.

As these are large-scale, resource intensive trials it was not possible to include sufficient true statistical replication in the study design. The difficulty of including replication in the design of large honey bee field trials is confirmed in the EPPO guidance document for evaluating the side effect of pesticides on honey bees [15], which states that although replication is desirable it is not feasible because of the requirements for separation (of the treatment and control fields). According to this guidance, individual hives are not replicates, and treatment effects should be considered on a plot-by-plot basis. Therefore expert judgment is needed to assess the biological significance of any effects seen in the context of each colony and the test conditions [15]. Colony results for each trial site presented in the results section and are reported as mean values of 6 hives. Error bars are not given as the treatment unit is the site plot not the hive. Hence it was considered inappropriate to show such error bars on the graphs presented [16]. In the analysis of the data, it was important to keep the maize and oilseed rape data separate as these two crops have different agronomy and thus different timings and durations of assessment were necessary. Also the bee exposure of these two crops differs as oil seed rape produces both pollen and nectar whilst maize only produces pollen. Despite the extensive amount of data generated, the fundamentals of the experimental design are such that, when it comes to making formal statistical comparisons between treatments, the only true replication is at the field level. Therefore, in terms of independent experimental units, the design for maize comprises two treatments × three locations, while that for oilseed rape comprises two treatments × two locations. Whilst it would, in principle, be possible to carry out a formal statistical analysis for both the maize and the oilseed rape data, the number of error degrees of freedom would be just two for maize and one for oilseed rape, and in practice an analysis based on so few degrees of freedom would be of no real value. Specifically, such an analysis would lack the power to detect anything other than very large treatment effects, and it is clear from a simple inspection of the results that no large treatment effects were present. Therefore a formal statistical analysis was not conducted because this would be potentially misleading.

## Results

### Residues in Plants, Pollen, Nectar and Bee Products Study

**Figure 2:**
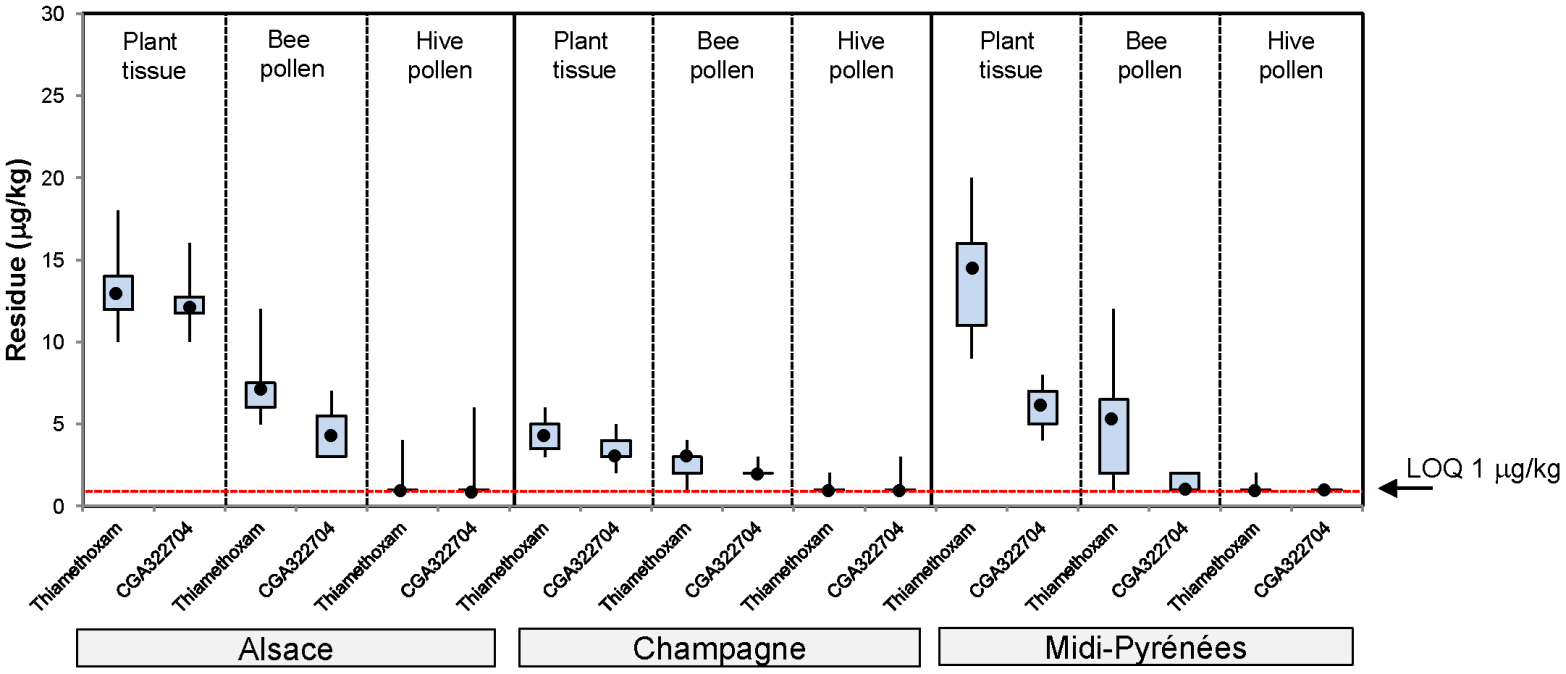
Thiamethoxam and CGA322704 residue levels in plant tissue, bee pollen and bee bread stored in cells from the first year of seed treated maize exposure in tunnels. The plot shows the median (•), upper and lower quartile and range values.

**Figure 3:**
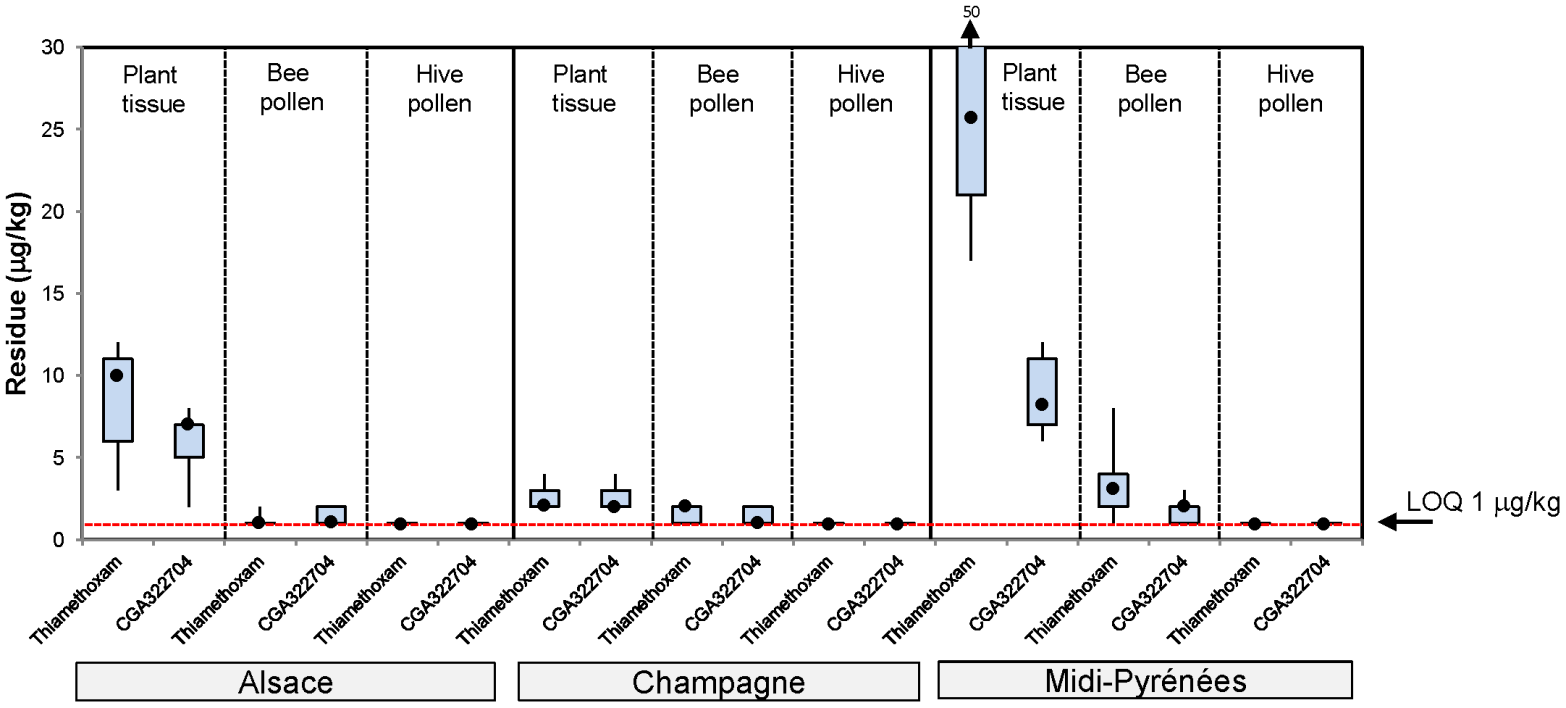
Thiamethoxam and CGA322704 residue levels in plant tissue, bee pollen and bee bread stored in cells from a two year seed treated maize rotation exposure in tunnels. The plot shows the median (•), upper and lower quartile and range values.

**Figure 4:**
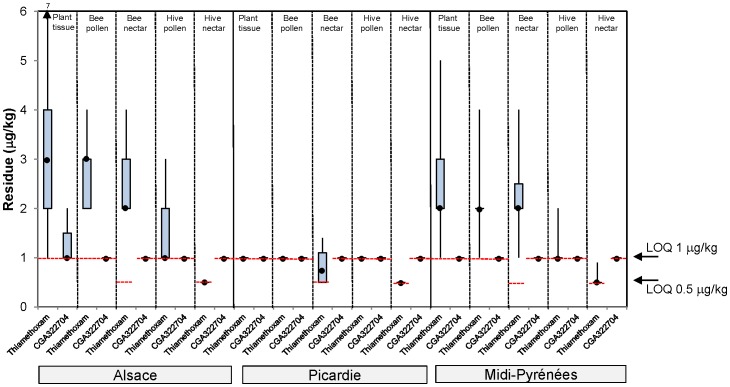
Thiamethoxam and CGA322704 residue levels in plant tissue, bee pollen, bee nectar, bee bread stored in cells and hive nectar from seed treated oilseed rape exposure in tunnels. The plot shows the median (•), upper and lower quartile and range values.

**Figure 5:**
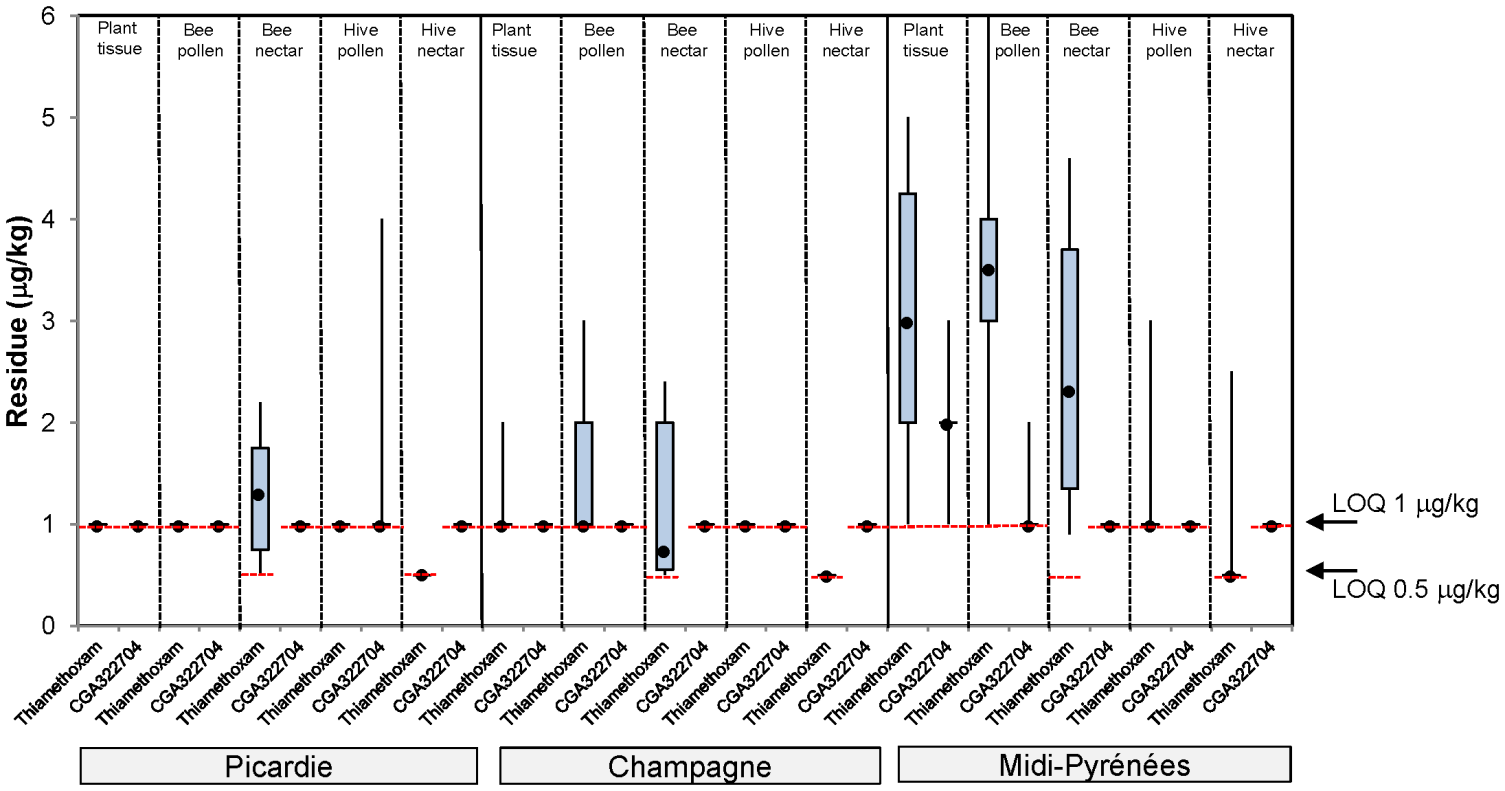
Thiamethoxam and CGA322704 residue levels in plant tissue, bee pollen, bee nectar, bee bread stored in cells and hive nectar from a seed treated oilseed rape following seed treated barley rotation exposure in tunnels. The plot shows the median (•), upper and lower quartile and range values.

The residue results from first year maize trials are shown in Fig. 2, from a two year maize rotation in Fig. 3, from 1 year oilseed rape in Fig. 4 and from oilseed rape following barley rotation in Fig. 5. A summary of the residue results from all the trials is provided in Table S1.

In first year maize in 2005, the median thiamethoxam residue levels in plant tissue collected at flowering ranged between 4 µg/kg in Champagne to 14 µg/kg in Midi-Pyrénées (Fig. 2). Data on residue levels in plant tissue are reported to provide a complete picture and potentially useful information as a predictor for pollen and nectar (in oilseed rape) levels. The corresponding median residue levels in pollen collected from foraging bees were always found to be lower than plant tissue, ranging from 3 µg/kg in Champagne to 7 µg/kg in Alsace. Residue levels of thiamethoxam analysed in bee bread stored in cells were lower than or equal to pollen collected from foraging bees, being at or below the 1 µg/kg LOQ. Additionally, the median residue levels of the metabolite CGA322704 were always lower than or equal to the parent thiamethoxam in all matrices tested, ranging from 3 to 12 µg/kg in plant tissue and 1 to 4 µg/kg in bee pollen.

In second year maize rotation in 2006, the median thiamethoxam residue levels in plant tissue collected at flowering ranged between 2 µg/kg in Champagne to 26 µg/kg in Midi-Pyrénées (Fig. 3). The corresponding median residue levels in pollen collected from foraging bees were always found to be lower than plant tissue, ranging from 1 µg/kg in Alsace to 3 µg/kg in Midi-Pyrénées. Residue levels of thiamethoxam analysed in bee bread stored in cells were lower than or equal to pollen collected from foraging bees, being at or below the 1 µg/kg LOQ. Additionally, the median residue levels of the metabolite CGA322704 were always lower than or equal to the parent thiamethoxam in all matrices tested ranging from 2 to 8 µg/kg in plant tissue and 1 to 2 µg/kg in bee pollen.

In the 1 year oilseed rape trials, the median thiamethoxam residue levels in plant tissue collected at flowering ranged between <1.0 µg/kg in Picardie to 3 µg/kg in Alsace (Fig. 4). The corresponding median residue levels in pollen collected from foraging bees was similar to plant tissue, ranging from <1.0 µg/kg in Picardie to 3 µg/kg in Alsace. The corresponding median residue levels in nectar collected from foraging bees was lower than or equal to plant tissue, ranging from 0.65 µg/kg in Picardie to 2 µg/kg in Alsace and Midi-Pyrénées. Residue levels of thiamethoxam found in bee bread stored in cells were lower than or equal to pollen collected from foraging bees, being at or below the 1 µg/kg LOQ. Residue levels of thiamethoxam found in hive nectar were lower than or equal to nectar collected from foraging bees, being at or below the 0.5 µg/kg LOQ. Additionally, the median residue levels of the metabolite CGA322704 were always lower than or equal to the parent thiamethoxam in all matrices tested, being at or below the 1.0 µg/kg LOQ in all matrices.

In the oilseed rape following treated barley rotations, median residues in plants at flowering were between <1 and 3 µg/kg, whereas median residue levels collected from foraging bees ranged from being at or below the LOQ (1.0 µg/kg) to 3.5 µg/kg in pollen and 0.7 µg/kg to 2.4 µg/kg in nectar (Fig. 5). In all oilseed rape trials residue levels in the bee bread stored in cells and hive nectar were consistently equal to or lower than nectar or pollen collected from bees, with median thiamethoxam residues being at or below the LOQ (1.0 and 0.5 µg/kg, respectively). As with maize, the median residue levels of the metabolite CGA322704 were always lower than or equal to the parent thiamethoxam in all matrices tested ranging from <1 to 2 µg/kg in plant tissue and <1 µg/kg for both bee pollen and bee nectar.

Residue levels of thiamethoxam and CGA322704 in hive wax and royal jelly were always below the LOQ (1.0 µg/kg) in both maize and oilseed rape trials at all but one location. The exception was in Midi-Pyrénées where oilseed rape followed barley. In this isolated case thiamethoxam residues up to 0.9 µg/kg were found in wax during the exposure period. Continued sampling of wax and other matrices in this trial showed residues were subsequently all below the LOQ indicating residues of thiamethoxam were not persistent in the wax.

### Multiple Exposure Bee Field Study

The results from the residue analysis conducted to establish the level of exposure in the multiple exposure trials are summarized in Table 1, showing the median and range of values determined. Median residues of both thiamethoxam and CGA322704 in whole plant material for both maize and oil seed rape were generally higher than reported for pollen and nectar, ranging from <1 to 8.5 µg/kg for thiamethoxam and <1 to 5.5 µg/kg for CGA322704. Residues of thiamethoxam were detected in the pollen collected from bees at all three maize sites (ranging from <1–2 µg/kg), in pollen and nectar collected from bees at the Alsace oilseed rape site and in nectar at the Picardie site (ranging from <0.5–3 µg/kg). Residues of the primary metabolite CGA322704 in pollen and nectar followed a similar pattern to thiamethoxam for both maize and oilseed rape but were generally lower. Therefore, honey bees at all 3 maize sites were clearly exposed to residues of thiamethoxam and its metabolite CGA322704, and to thiamethoxam at both oilseed rape sites. Levels were similar to those found in the comprehensive residue study also reported in this paper. Therefore these residues are representative of the exposure that would occur under field conditions from the use of thiamethoxam on those crops at the maximum recommended field rate in Europe.

**Table 1:** Median residue values (and range in parentheses) found in plants, pollen and nectar collected from forager bees in maize and oilseed rape from 2005 to 2009 in the multi-exposure study.

Crop	Location	Sample type	Median Thiamethoxam residue^1^ (µg/kg)		Median CGA322704 residue^1^ (µg/kg)	
			LOQ = 1 µg/kg		LOQ = 1 µg kg	
			Control	Treated	Control	Treated
Maize	Alsace	Plant	<1	5 (1.3–24)	<1	4 (1.9–10)
		Pollen (bee)	<1	<1 (<1–2)	<1	<1 (<1–2)
Maize	Lorraine	Plant	<1	4.5 (3–6)	<1	4.5 (4–6)
		Pollen (bee)	<1	<1 (<1–1)	<1	<1 (<1–2)
Maize	Aveyron	Plant	<1	8.5 (6–10)	<1	5.5 (5–8)
		Pollen (bee)	<1	<1 (<1–1)	<1	1 (<1–2)
Oilseed rape	Alsace	Plant	<1	<1 (<1–2)	<1	1 (<1–1)
		Pollen (bee)	<1	1 (<1–1)	<1	<1
		Nectar (bee)	<0.5^2^	1.7^2^ (<0.5–3)	<1	<1
Oilseed rape	Picardie	Plant	<1	<1	<1	<1
		Pollen (bee)	<1	<1	<1	<1
		Nectar (bee)	<0.5^2^	0.7^2^	<1	<1

1 Range of residue values given in parentheses.

2 LOQ = 0.5 µg/kg.

The biological results from the Alsace trial site with oilseed rape are shown throughout this paper as an example of typical data obtained during the investigation. The data from this site were specifically chosen as this was the site with the highest reported residues of thiamethoxam and its metabolite in nectar. The biological results from the other trial sites with maize and oilseed rape were consistent across all sites throughout the trial and are shown in the Supporting Information.

**Figure 6:**
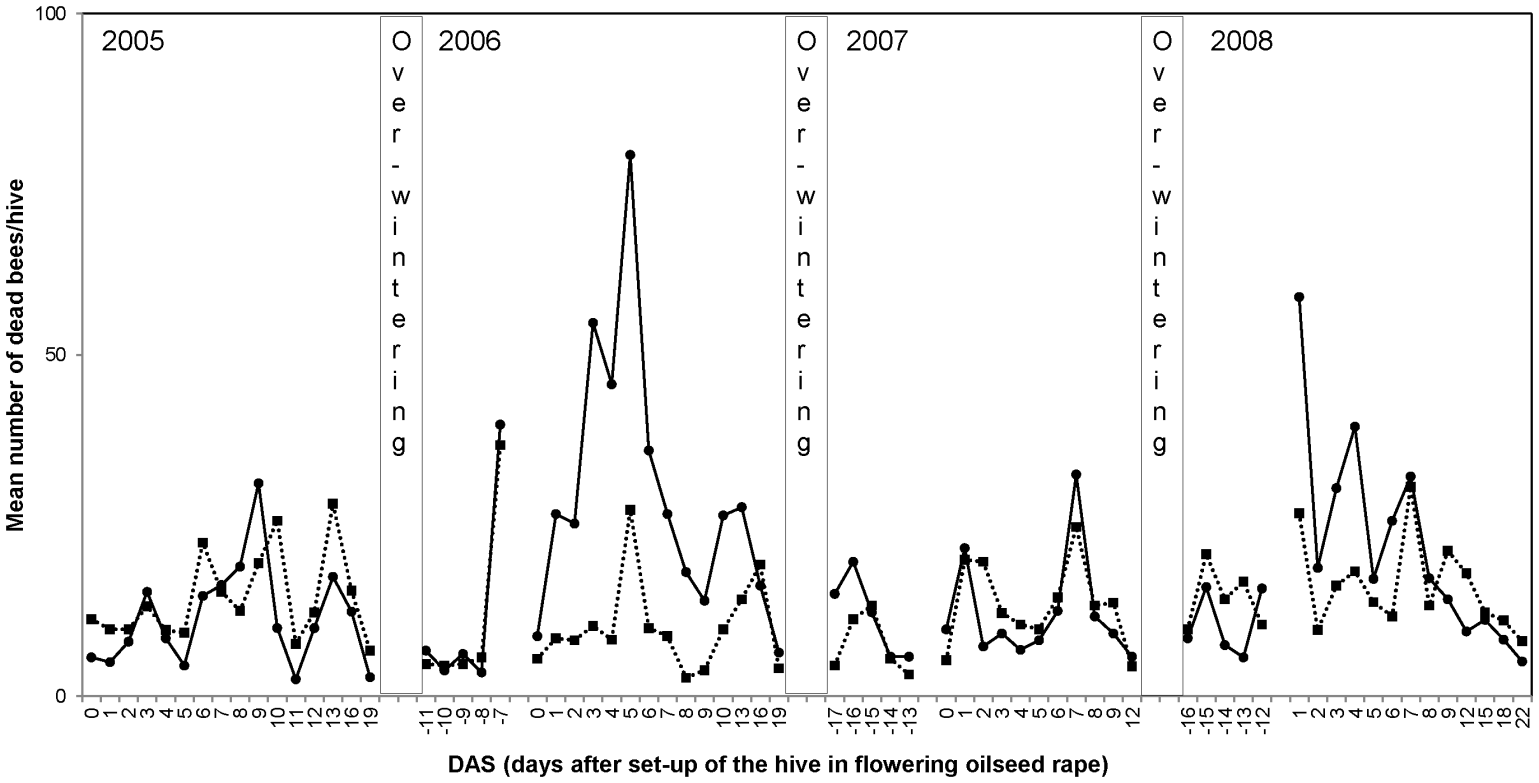
Mean number of dead bees per hive per day collected in the dead bee traps and on linen sheets in front of the hives in treated (dashed line) and control (solid line) oilseed rape fields in the Alsace region of France.

The mean number of dead bees in front of the colonies (dead bee traps and linen sheets) placed adjacent to treated and control oilseed rape fields in the Alsace region of France is shown in Fig. 6. Dead bee results from the other trials in maize and oilseed rape are shown in Figs. S1 to S4. The number of dead bees was considered to be low in all treated and control hives throughout the period of the trial and, on average, dead bee levels were similar in treatment and control colonies for both maize and oilseed rape at all sites. Short-term increases in the number of dead bees were recorded for colonies exposed to both treated and control crops, mainly at the start of the exposure period after the brood assessments were carried out, or after transport of the colonies to the test fields. Typically, the average number of dead bees for colonies exposed to both control and treated crops was below 100 per colony per day (with the exception of Aveyron where average number of dead bees peaked at around 230 in the control). Linen sheets spread out in the control and treated fields recorded very low numbers of dead bees throughout the trial at all five locations.

**Figure 7:**
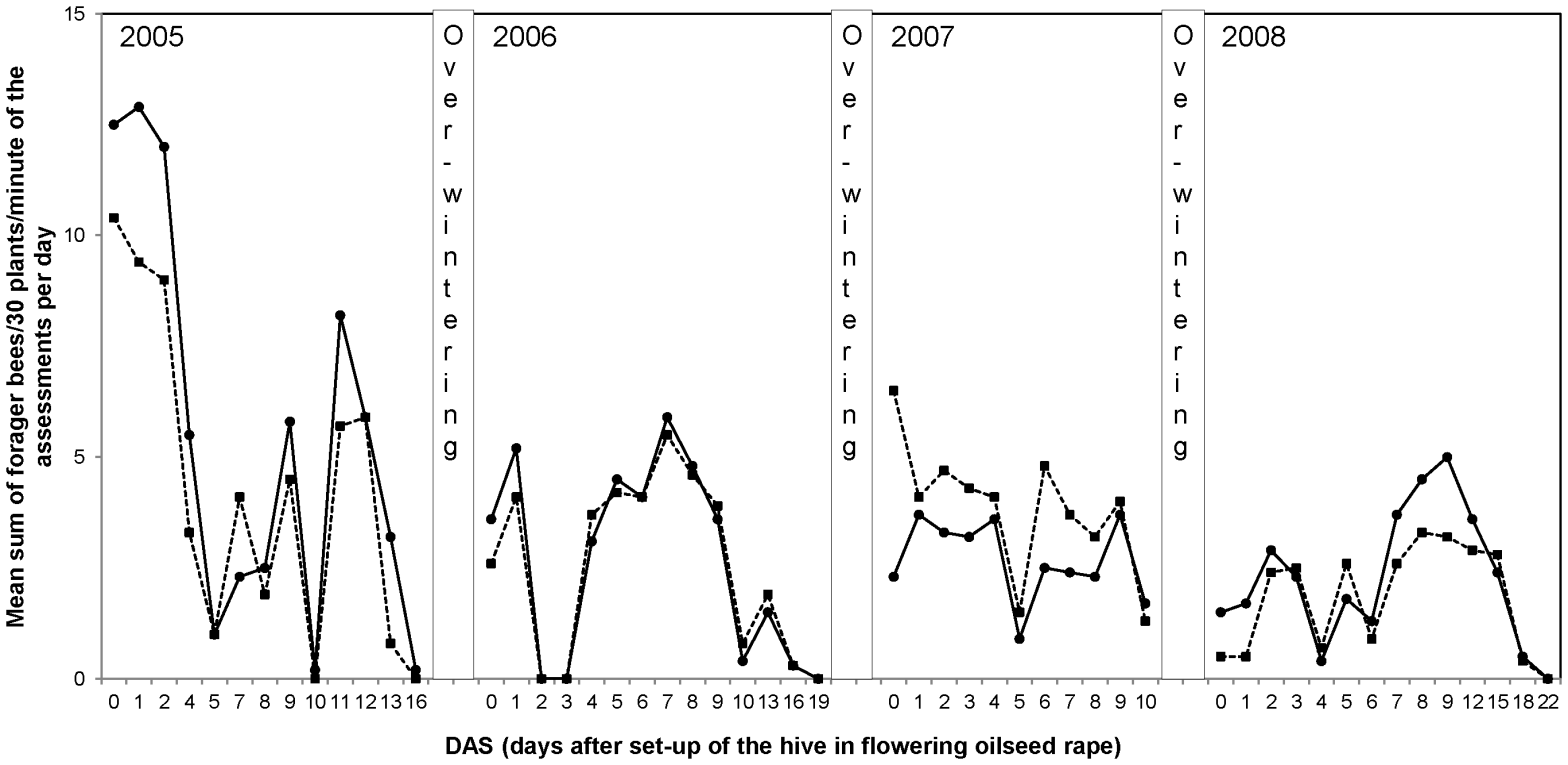
Mean number of forager bees per m^2^ flowering rape in treated (dashed line) and control (solid line) fields during the time of exposure in the Alsace region of France.

Figure 7 shows the foraging activity of bees in oilseed rape treated and control fields at the Alsace trial site. Foraging activity data from the other trial sites in maize and oilseed rape are shown in Figs. S5 to S8. Over the four years of assessments, on average, the foraging activity was similar between treatment and control colonies at all sites. At the end of the observation period the flight intensity decreased in both the treatment and control fields because the crop was coming to the end of its flowering period. Analysis of pollen loads collected at the hive entrance from forager bees showed the mean proportion of oilseed rape pollen on individual sampling days varied from 15 to 64% in the treatment and 7 to 75% in the control over the study period. In the maize trials, pollen collected on individual sampling days at the hive entrance varied from 0 to 82% in the treatment and 0 to 55% in the control over the 4 year study period. The variation in proportion of maize pollen collected in the pollen loads is not surprising as maize does not produce nectar and is considered less attractive to bees in comparison to oilseed rape. Flower surveys of the surrounding area during the exposure period showed limited alternative forage was available.

**Figure 8:**
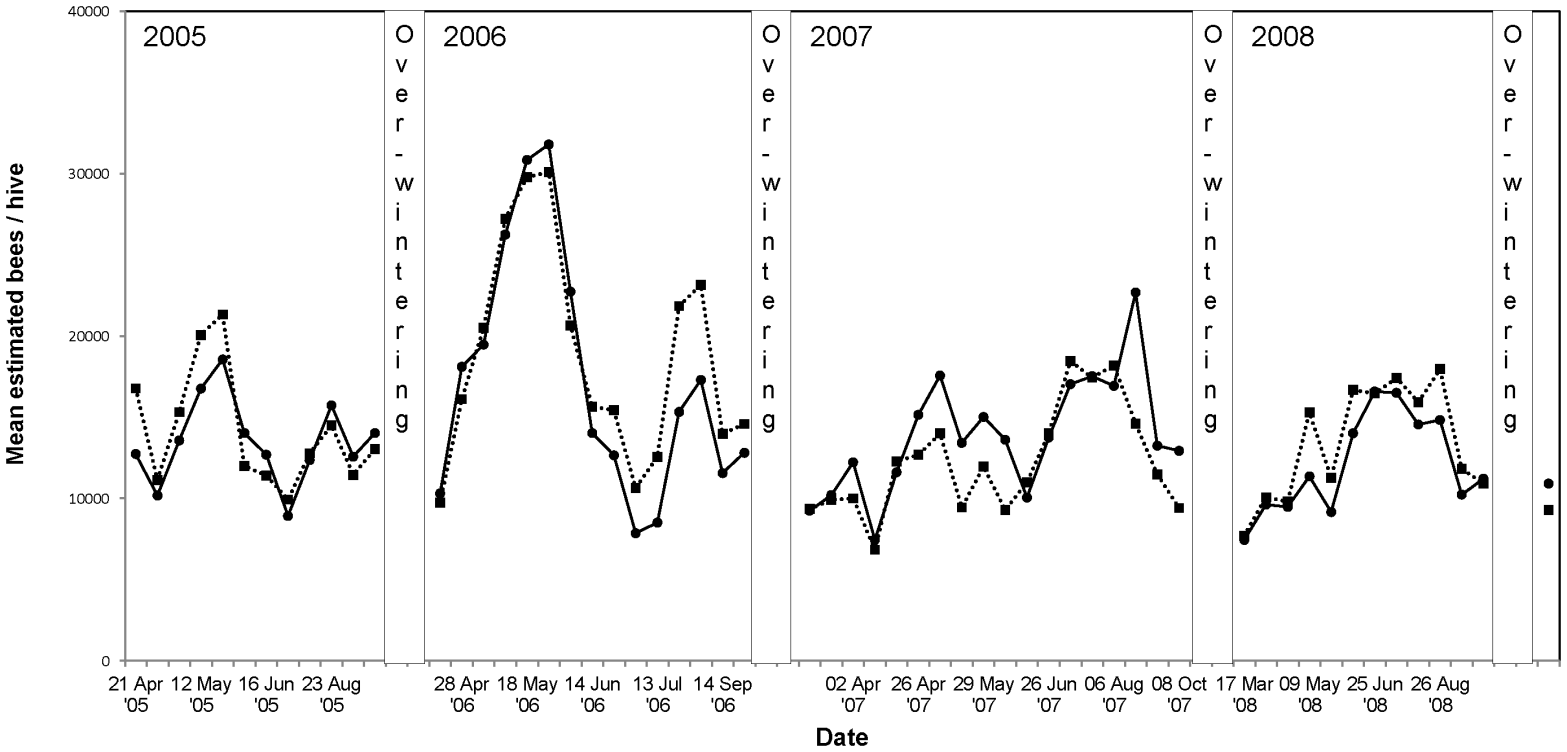
Strength of honey bee colonies exposed to treated (dashed line) and control (solid line) oilseed rape fields in the Alsace region of France during the four years of observations including the last overwintering.

Colony strength (represented as the number of bees per colony) exposed to treated or control oilseed rape fields in the Alsace region of France is shown in Fig. 8. The colony strength data from the other trial sites in maize and oilseed rape are shown in Figs. S9 to S12. The strength of both the control and treatment colonies showed approximately the same tendency to increase or decrease over the duration of the trial in both oilseed rape and maize. In spring the colonies were increasing their size and strength because of an increased food supply at the monitoring site or in the test fields. After bee colony separation or size reduction at the end of the exposure period, a decrease in the number of bees in both the treated and control colonies was recorded. After colonies had produced a fertile queen the egg laying started again and therefore the number of bees built up again in June or July of each year. At the end of the observation period in autumn the strength of the control and treatment colonies decreased again because of the natural decrease in egg laying before winter. At the end of the overwintering period at all trial sites, all control and treated colonies in both maize and oilseed rape trials were, on average, approximately the same strength.

**Figure 9:**
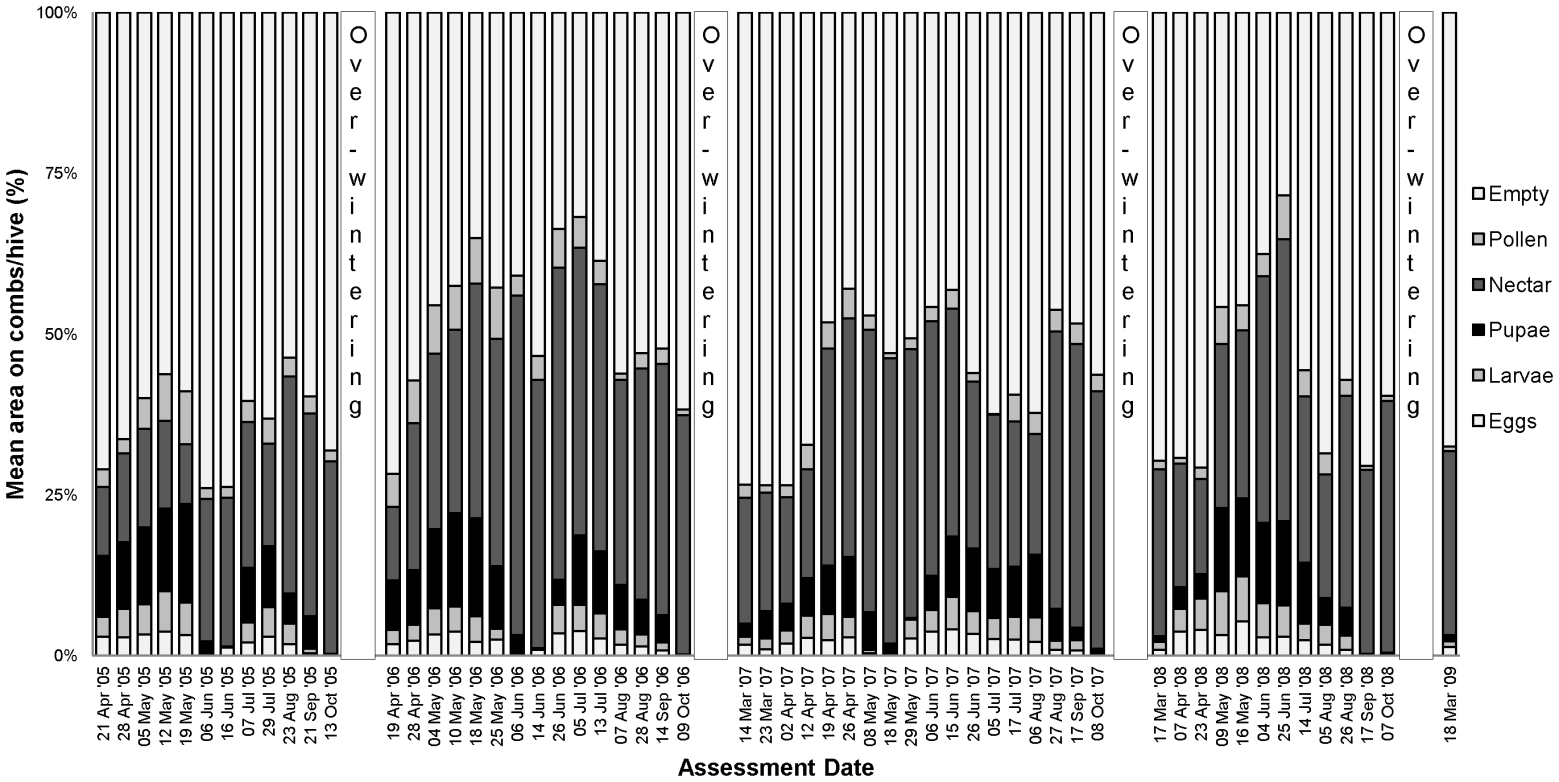
Mean area on combs (%) of brood (eggs, larvae and pupae) and food (nectar and pollen) of 6 colonies exposed to treated oilseed rape in the Alsace region of France over 4 years.

**Figure 10:**
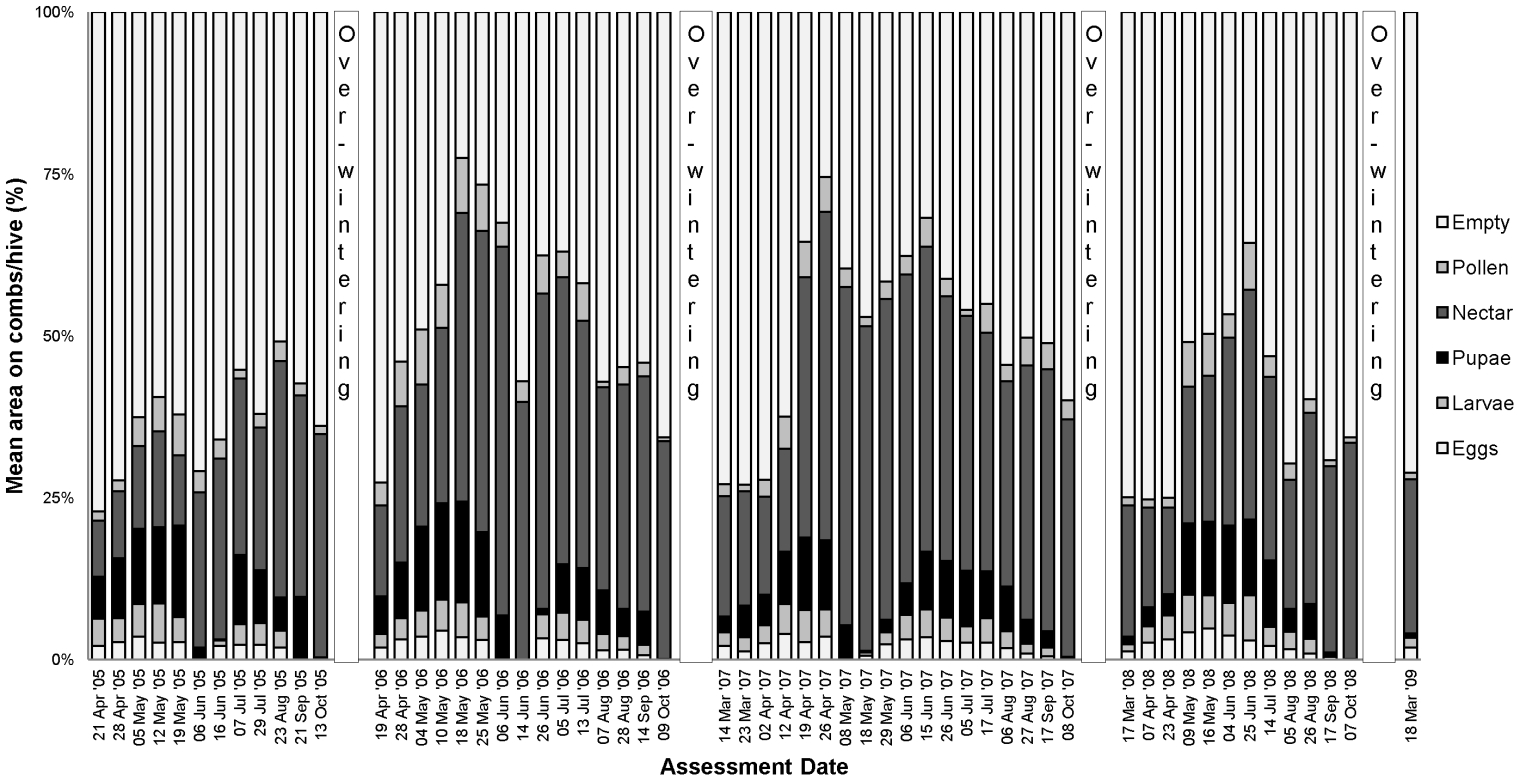
Mean area on combs (%) of brood (eggs, larvae and pupae) and food (nectar and pollen) of 6 colonies exposed to control oilseed rape in the Alsace region of France over 4 years.

The percentage comb coverage of brood (eggs, larvae and pupae) and food (nectar and pollen) for colonies exposed to oilseed rape at the Alsace site are shown in Fig. 9 for the treated colonies and Fig. 10 for the control. The brood and food area graphs from the other trial sites in maize and oilseed rape are shown in Figs. S13 to S20. Colonies showed the same brood and food development patterns in the control and treatment groups for both crops at all sites. The amount of brood decreased up to the last assessment each year because of a natural decrease in the egg laying and a lower amount of natural food source before winter. In spring each year, colonies increased their egg laying and food stores with the increasing availability of food from flowering plants. The cycle of brood development throughout the year was found to be in line with normal honey bee biology [17] in both treatment and control colonies. After bee colony separation where combs and the old queen were removed to form a nucleus, colonies showed a decrease in egg laying represented by decreased brood area coverage. When colonies had produced a new fertile queen, the egg laying started again and the amount of brood on the combs increased in both the control and treatment groups.

During the four year study period, some colonies were found to produce male brood only indicating the absence of a healthy egg-laying queen or some colonies were lost due to disease, with a mean loss ranging from 0 to 2.7 colonies per year across all five sites (total of 60 colonies) and with similar losses observed between treated and control sites (Table 2). On such occasions, the queen was replaced according to standard beekeeping practice or the colony was replaced with the nucleus colony produced from the previous splitting event, thus allowing the trial to continue. The frequency of queen replacement and colony loss was as would be expected with this number of colonies over a four year period and considered to be in line with normal beekeeping practice [17].

**Table 2:** Details of colony losses throughout the multiple exposure bee field trial in maize and oilseed rape.

Crop	Location	Date, number and cause of colony losses	
		Control	Treated
Maize	Alsace	September 2008, single colony lost dueto male brood only	March 2008, single colony lost due to male brood only
		March 2009, single colony lost dueto male brood only	May 2008, single colony lost due to male brood only
			March 2009, single colony lost due to male brood only
			March 2010, single colony lost due to male brood only
Maize	Lorraine	May 2008, 2 colonies lost dueto male brood only	July 2007, 4 colonies lost due to male brood only
		April 2009, single colony lost dueto male brood only	April 2008, 2 colonies lost due to male brood only
		September 2009, single colonylost due to male brood only	
		March 2010, single colonylost due to male brood only	
Maize	Aveyron	August 2007, single colony destroyeddue to AFB	September 2006, single colony destroyed due to AFB
		April 2009, single colony lostdue to male brood only	March 2007, 2 colonies destroyed due to AFB
		August 2009, 2 colonies lostdue to male brood only	May 2007, single colony lost due to male brood only
		October 2009, single colonylost due to male brood only	August 2007, single colony lost due to male brood only
		March 2010, 5 colonieslost due to male brood only	May 2008, 2 colonies lost due to male brood only
			September 2008, 1colony lost due to male brood only
			March 2010, 3 colonies lost due to male brood only
Oilseed rape	Alsace	March 2008, single colony lostdue to male brood only	
		March 2009, single colony lost due to male brood only	
Oilseed rape	Picardie	June 2005, single colony lostdue to male brood only	March 2007, 3 colonies lost due to male brood only
		March 2008, single colony lostdue to male brood only	

**Figure 11:**
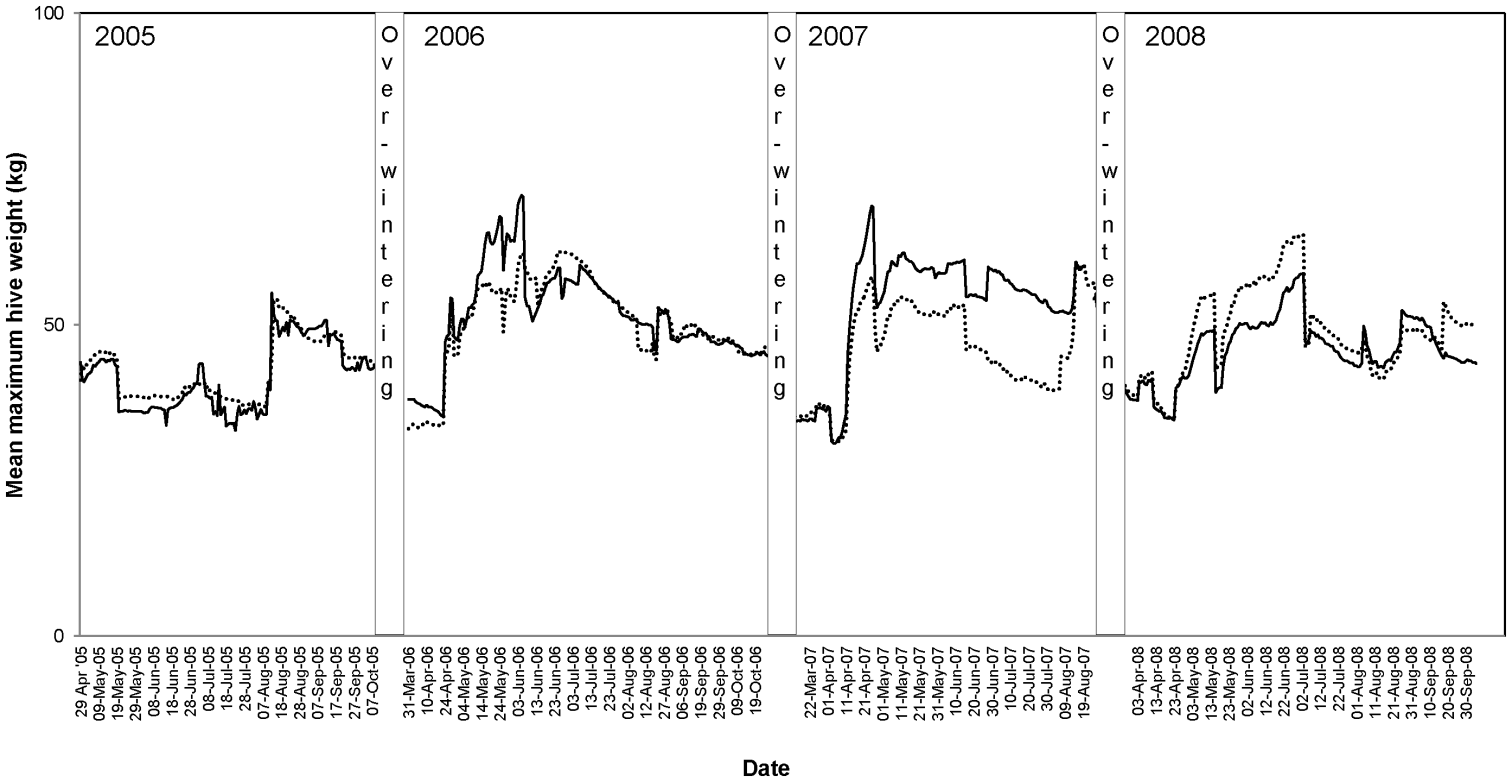
Mean hive weight (kg) during time of assessments of colonies in treated (dashed line) and control (solid line) oilseed rape fields in the Alsace region of France.

The mean weight of colonies exposed to treated and control oilseed rape fields in the Alsace region of France are shown in Fig. 11. The hive weight graphs from the other trial sites in maize and oilseed rape are shown in Figs. S21 to S24. In general, the mean weight increase and decrease of the colonies exposed to treated and control crops was similar in both maize and oilseed rape crops at each trial site. Some changes in mean weights over time were observed, though these were predominantly due to different bee keeping activities (e.g. bee colony separation, removing of combs, feeding of colonies).

## Discussion

Neonicotinoids are insecticides and as such are toxic to bees in laboratory studies with LD_50_ values typically between 3 ng a.s./bee and 39 µg a.s./bee depending principally on the route of exposure, age of bee and class of neonicotinoid [18]. Various studies where bees have been dosed in the laboratory or used artificial feeders have shown sublethal effects occur following exposure to neonicotinoids, for example causing impairment of honey bee homing behaviour [19], foraging behavior [20], or reducing colony growth and queen production in bumble bees [21], [22]. However, these experiments on sublethal effects have often been conducted under artificial exposure conditions or at exposure levels much higher than would occur under field conditions following normal agronomic use [23]. For example, in the paper by Schneider *et al.* [20], no adverse effects were observed when bees were exposed to imidacloprid or clothianidin at field-relevant doses; effects on foraging activity were only recorded at higher doses.

The detailed residue data reported in this paper provide yet further confirmation that residues of thiamethoxam and its primary metabolite CGA322704 in pollen and nectar collected from bees exclusively foraging in flowering thiamethoxam seed treated maize and oilseed rape are typically low, with median values between <1 and 7 µg/kg for pollen) and <0.5 and 4 µg/kg for nectar. In addition, residues in hive collected bee bread stored in cells and hive nectar samples were consistently lower than residues in pollen and nectar samples collected from bees, indicating dilution and/or degradation of residue in the hive following collection. There was no reported increase in pollen and nectar residues for thiamethoxam seed treated oilseed rape when drilled as a rotation immediately following harvest of a thiamethoxam seed treated barley crop or for thiamethoxam treated maize in two year rotation. This indicates no residue carry-over or accumulation from typical crop rotation practices. Similar residue levels (i.e. 0.9 µg/kg in pollen and 0.7 µg/kg in honey) have also been reported for imidacloprid seed treatment uses on a range of bee attractive crops from a survey of 24 sites and 120 colonies over a three year period [24], [25], [26].

There have been very few in-use field studies published that have investigated long-term and repeated exposure of honey bee colonies to neonicotinoid insecticides that included the sensitive overwintering period. Faucon *et al*. [27] exposed colonies to imidacloprid in saccharose syrup at doses of 0.5 and 5.0 µg/L during the summer and monitored their development and survival until the end of the season and then again the following spring after the overwintering period. Repeated feeding with syrup supplemented with imidacloprid did not cause any immediate or delayed adverse effects until the end of the observation period i.e. until the beginning of the following spring. Cutler and Scott-Dupree [28] placed colonies in the middle of 1 ha canola (oilseed rape) fields planted with clothianidin-treated seed at a high rate, equivalent to 32 g a.s./ha. To assess long-term effects, these colonies were intensely monitored throughout the 3-week flowering period and subsequently whilst in an apiary over winter through to the following spring. Again no effects on honey production, brood production, number of adult workers, overwinter survival and overall health were evident between colonies from clothianidin treated and control fields. The authors concluded that field exposure to clothianidin treated canola seed presented negligible risk to honey bees.

The results reported here from the large scale field studies also show no evidence of detrimental effects on colonies that were repeatedly exposed over a four-year period to thiamethoxam residues in pollen and nectar, following seed treatment of oilseed rape and maize. During the exposure period, when colonies were placed adjacent to treated and control fields, the number of dead bees, foraging behavior, colony strength, brood development and food storage levels were, on average, similar between treatment and control colonies. By monitoring the weight of colonies throughout the four-year period, it was evident that there were also no substantial losses of foraging bees exposed to treated crops in the field. Detailed examination of brood development throughout the year demonstrated that colonies exposed to the treated crop were also able to successfully overwinter and had a similar health status to the control colonies in the following spring. The results from these large-scale field trials provide a different conclusion to recent published studies examining mortality and sub-lethal effects following artificial exposure conditions [19], [21]. However, as can be seen from the pollen and nectar residue data reported in this study, this can be explained by the fact that exposure and hence risk to honey bees from systemic thiamethoxam residues in pollen and nectar following seed treatments is low under real in-use field conditions.

Field trials of this magnitude conducted over numerous years are complex and difficult to conduct. Finding suitable trial sites with sufficient distance between the control and treatment plots, which are also separated from other potentially confounding pesticide treated alternative foraging sites, is very challenging. Since conducting such trials with sufficient replication to allow robust statistical analysis is currently practically unfeasible, expert interpretation of the data by scientists with experience in undertaking such trials is considered vital. Furthermore, colony loss over a four year period can be high in these type of trials due to the loss of healthy egg-laying queens and background disease. This may also make interpretation of the data challenging. Risk assessment based on laboratory data alone, however, will not provide critical information on realistic exposure or behavior of bees following actual use of the pesticide under normal agronomical conditions. Therefore, while there can be limitations and challenges with regards to conducting and interpreting field trials, the information generated adds considerable value to our understanding of risk under real in-use field conditions.

Cresswell *et al*. [29] used Hill’s epidemiological ‘causality criteria’ to examine the literature evidence relating to the use of neonicotinoid insecticides as a cause of honey bee population decline. On the basis of theoretical criteria, the proposition that dietary exposure to neonicotinoids cause honey bee decline scored positively, thus it is not surprising these insecticides have been widely implicated as a possible cause. However, virtually all the circumstantial epidemiological evidence clearly contradicts this. The authors concluded that dietary exposure to neonicotinoids present in trace levels in pollen and nectar cannot be implicated in honey bee declines, but that gaps remain in our current knowledge. In this study, at realistic field exposures to pollen and nectar from the recommended use of thiamethoxam as a seed treatment on maize and oil seed rape, no detrimental effects on colony survival and overwintering success were reported. These results are consistent with the findings of Cresswell *et al*. [29], and contribute towards improving our understanding of exposure and risk to honey bees from the use of neonicotinoids as seed treatments under field conditions.

## Supporting Information

Figure S1 **Mean number of dead bees per hive per day collected in the dead bee traps and on linen sheets in front of the hives in treated (dashed line) and control (solid line) maize fields in the Alsace region of France from 2006 to 2009.**
(TIFF)

Figure S2 **Mean number of dead bees per hive per day collected in the dead bee traps and on linen sheets in front of the hives in treated (dashed line) and control (solid line) maize fields in the Lorraine region of France from 2006 to 2009.**
(TIFF)

Figure S3 **Mean number of dead bees per hive per day collected in the dead bee traps and on linen sheets in front of the hives in treated (dashed line) and control (solid line) maize fields in the Aveyron region of France from 2006 to 2009.**
(TIFF)

Figure S4 **Mean number of dead bees per hive per day collected in the dead bee traps and on linen sheets in front of the hives in treated (dashed line) and control (solid line) oilseed rape fields in the Picardie region of France from 2005 to 2008.**
(TIFF)

Figure S5 **Mean number of forager bees per m^2^ flowering maize in treated (dashed line) and control (solid line) fields during the time of exposure in the Alsace region of France from 2006 to 2009.**
(TIFF)

Figure S6 **Mean number of forager bees per m^2^ flowering maize in treated (dashed line) and control (solid line) fields during the time of exposure in the Lorraine region of France from 2006 to 2009.**
(TIFF)

Figure S7 **Mean number of forager bees per m^2^ flowering maize in treated (dashed line) and control (solid line) fields during the time of exposure in the Aveyron region of France from 2006 to 2009.**
(TIFF)

Figure S8 **Mean number of forager bees per m^2^ flowering oilseed rape in treated (dashed line) and control (solid line) fields during the time of exposure in the Picardie region of France from 2005 to 2008.**
(TIFF)

Figure S9 **Strength of honey bee colonies exposed to treated (dashed line) and control (solid line) maize fields in the Alsace region of France during the four years of observations including the last overwintering.**
(TIFF)

Figure S10 **Strength of honey bee colonies exposed to treated (dashed line) and control (solid line) maize fields in the Lorraine region of France during the four years of observations including the last overwintering.**
(TIFF)

Figure S11 **Strength of honey bee colonies exposed to treated (dashed line) and control (solid line) maize fields in the Aveyron region of France during the four years of observations including the last overwintering.**
(TIFF)

Figure S12 **Strength of honey bee colonies exposed to treated (dashed line) and control (solid line) oilseed rape fields in the Picardie region of France during the four years of observations including the last overwintering.**
(TIFF)

Figure S13 **Mean area on combs (%) of brood (eggs, larvae and pupae) and food (nectar and pollen) of 6 colonies exposed to treated maize in the Alsace region of France over 4 years.**
(TIFF)

Figure S14 **Mean area on combs (%) of brood (eggs, larvae and pupae) and food (nectar and pollen) of 6 colonies exposed to control maize in the Alsace region of France over 4 years.**
(TIFF)

Figure S15 **Mean area on combs (%) of brood (eggs, larvae and pupae) and food (nectar and pollen) of 6 colonies exposed to treated maize in the Lorraine region of France over 4 years.**
(TIFF)

Figure S16 **Mean area on combs (%) of brood (eggs, larvae and pupae) and food (nectar and pollen) of 6 colonies exposed to control maize in the Lorraine region of France over 4 years.**
(TIFF)

Figure S17 **Mean area on combs (%) of brood (eggs, larvae and pupae) and food (nectar and pollen) of 6 colonies exposed to treated maize in the Aveyron region of France over 4 years.**
(TIFF)

Figure S18 **Mean area on combs (%) of brood (eggs, larvae and pupae) and food (nectar and pollen) of 6 colonies exposed to control maize in the Aveyron region of France over 4 years.**
(TIFF)

Figure S19 **Mean area on combs (%) of brood (eggs, larvae and pupae) and food (nectar and pollen) of 6 colonies exposed to treated oilseed rape in the Picardie region of France over 4 years.**
(TIFF)

Figure S20 **Mean area on combs (%) of brood (eggs, larvae and pupae) and food (nectar and pollen) of 6 colonies exposed to control oilseed rape in the Picardie region of France over 4 years.**
(TIFF)

Figure S21 **Mean hive weight (kg) during time of assessments of colonies in treated (dashed line) and control (solid line) maize fields in the Alsace region of France.**
(TIFF)

Figure S22 **Mean hive weight (kg) during time of assessments of colonies in treated (dashed line) and control (solid line) maize fields in the Lorraine region of France.**
(TIFF)

Figure S23 **Mean hive weight (kg) during time of assessments of colonies in treated (dashed line) and control (solid line) maize fields in the Aveyron region of France.**
(TIFF)

Figure S24 **Mean hive weight (kg) during time of assessments of colonies in treated (dashed line) and control (solid line) oilseed rape fields in the Picardie region of France.**
(TIFF)

Table S1 **Median thiamethoxam and CGA322704 residue values (and range in parentheses) found in plants, bee and hive pollen following exposure in maize in tunnels, and bee pollen and nectar and hive pollen and nectar following exposure in oilseed rape in tunnels.**
(DOCX)
