# Supplementary figures and images for: A Four-Year Field Program Investigating Long-Term Effects of Repeated Exposure of Honey Bee Colonies to Flowering Crops Treated with Thiamethoxam
Source: PLoS One. 2013 Oct 23;8(10):e77193. doi: 10.1371/journal.pone.0077193 (PMC3806756; doi:10.1371/journal.pone.0077193)

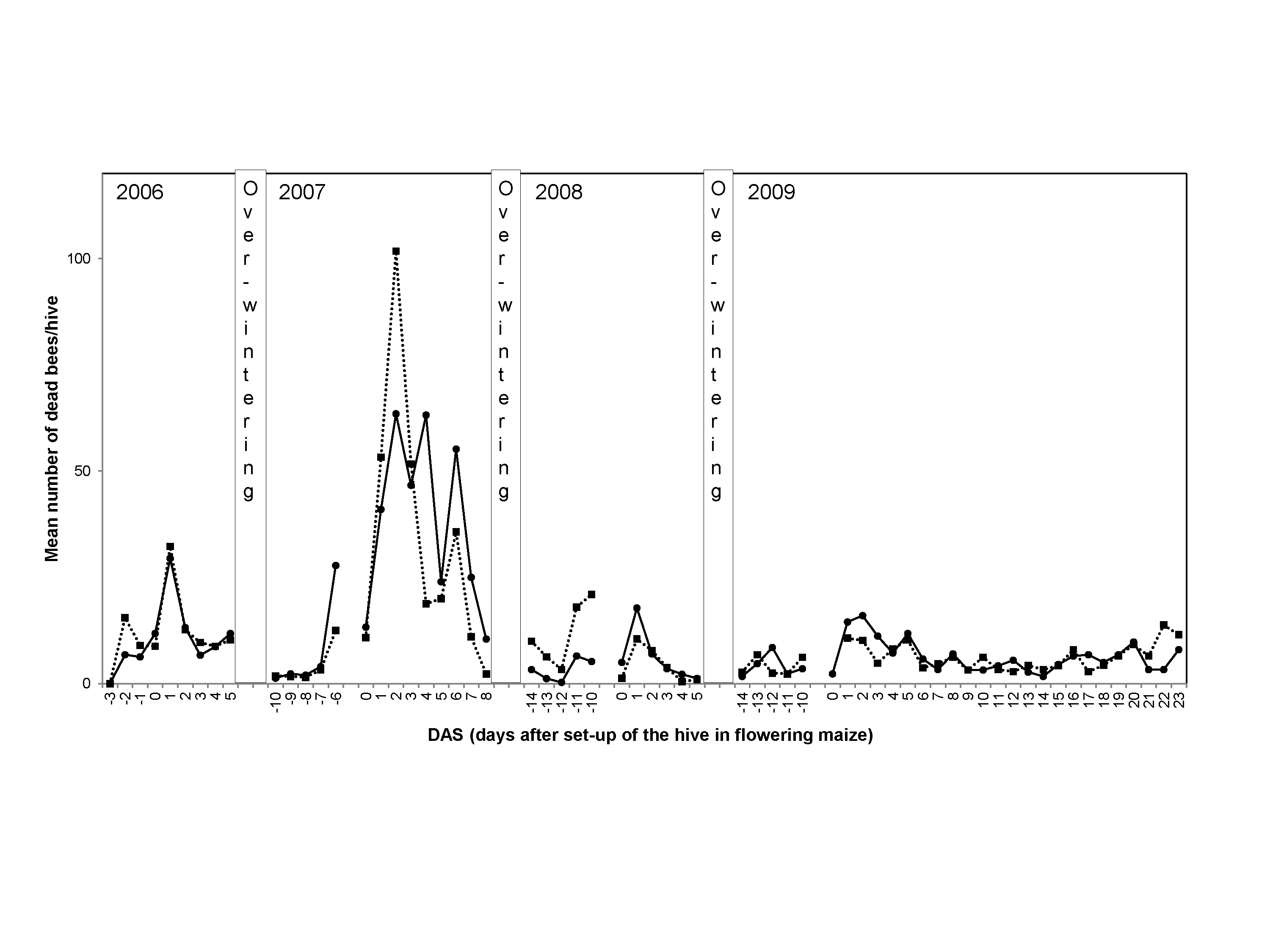

Supplement: Figure S1 — Mean number of dead bees per hive per day collected in the dead bee traps and on linen sheets in front of the hives in treated (dashed line) and control (solid line) maize fields in the Alsace region of France from 2006 to 2009. (TIFF) [file pone.0077193.s001.tiff]

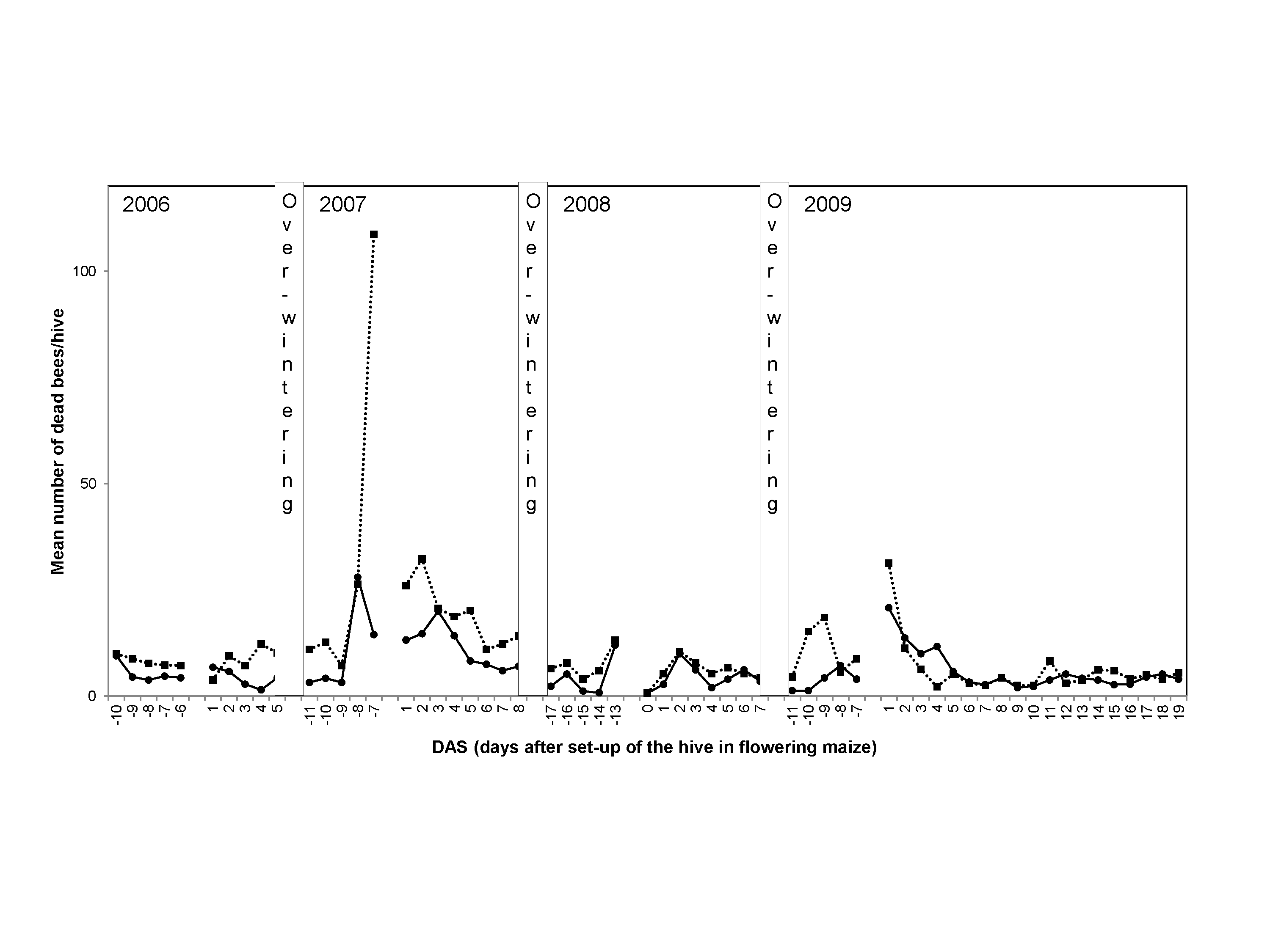

Supplement: Figure S2 — Mean number of dead bees per hive per day collected in the dead bee traps and on linen sheets in front of the hives in treated (dashed line) and control (solid line) maize fields in the Lorraine region of France from 2006 to 2009. (TIFF) [file pone.0077193.s002.tiff]

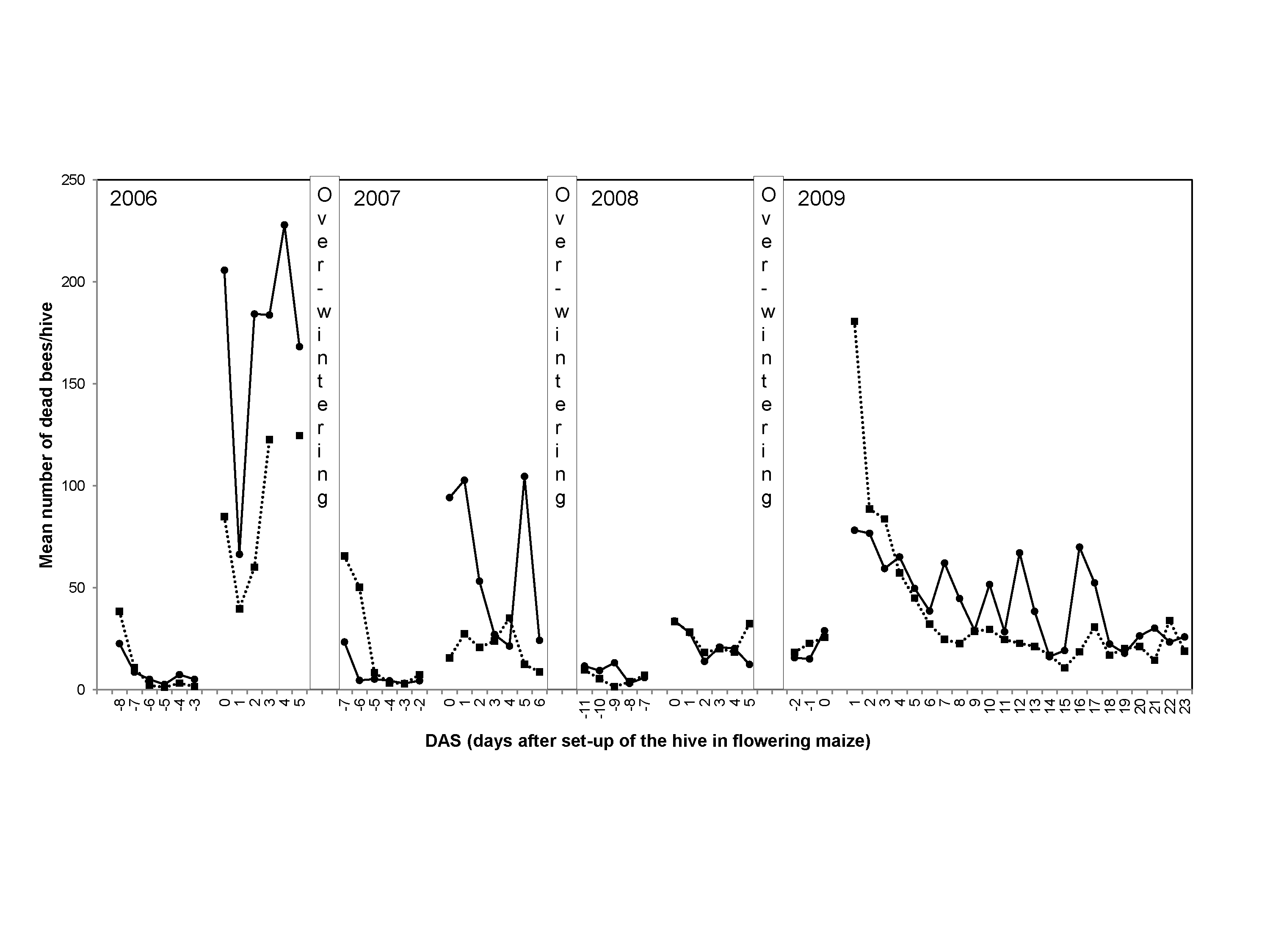

Supplement: Figure S3 — Mean number of dead bees per hive per day collected in the dead bee traps and on linen sheets in front of the hives in treated (dashed line) and control (solid line) maize fields in the Aveyron region of France from 2006 to 2009. (TIFF) [file pone.0077193.s003.tiff]

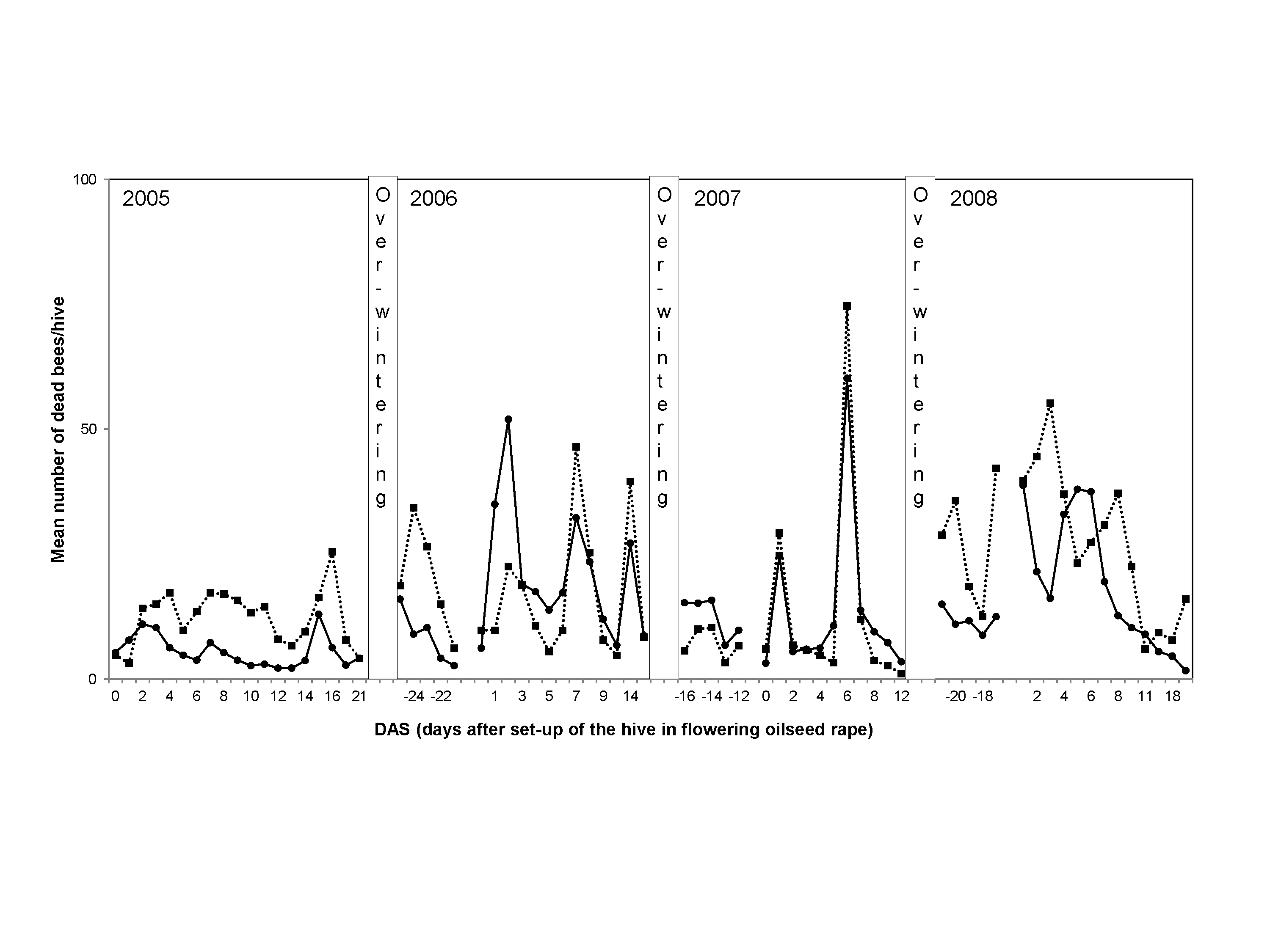

Supplement: Figure S4 — Mean number of dead bees per hive per day collected in the dead bee traps and on linen sheets in front of the hives in treated (dashed line) and control (solid line) oilseed rape fields in the Picardie region of France from 2005 to 2008. (TIFF) [file pone.0077193.s004.tiff]

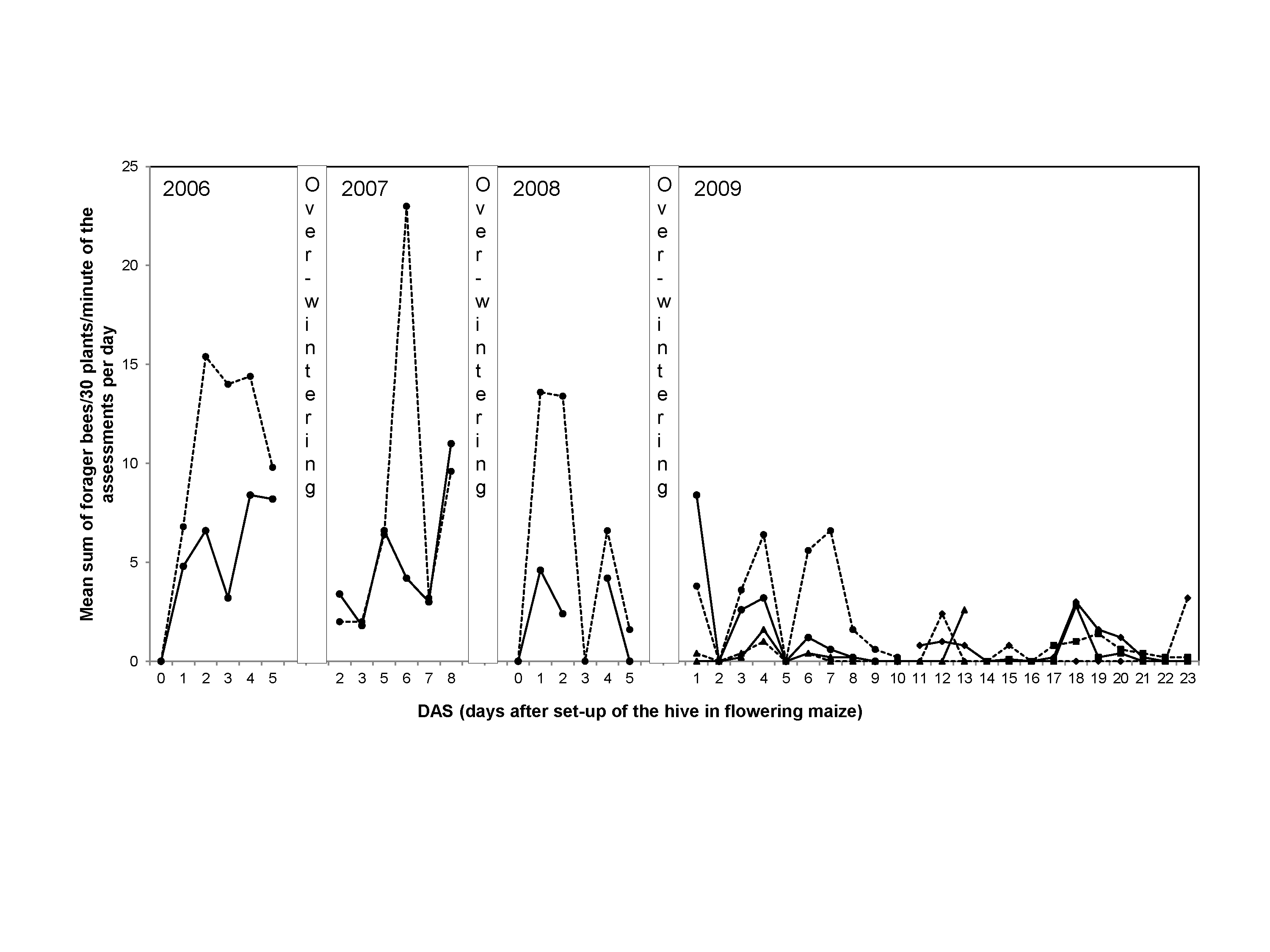

Supplement: Figure S5 — Mean number of forager bees per m2 flowering maize in treated (dashed line) and control (solid line) fields during the time of exposure in the Alsace region of France from 2006 to 2009. (TIFF) [file pone.0077193.s005.tiff]

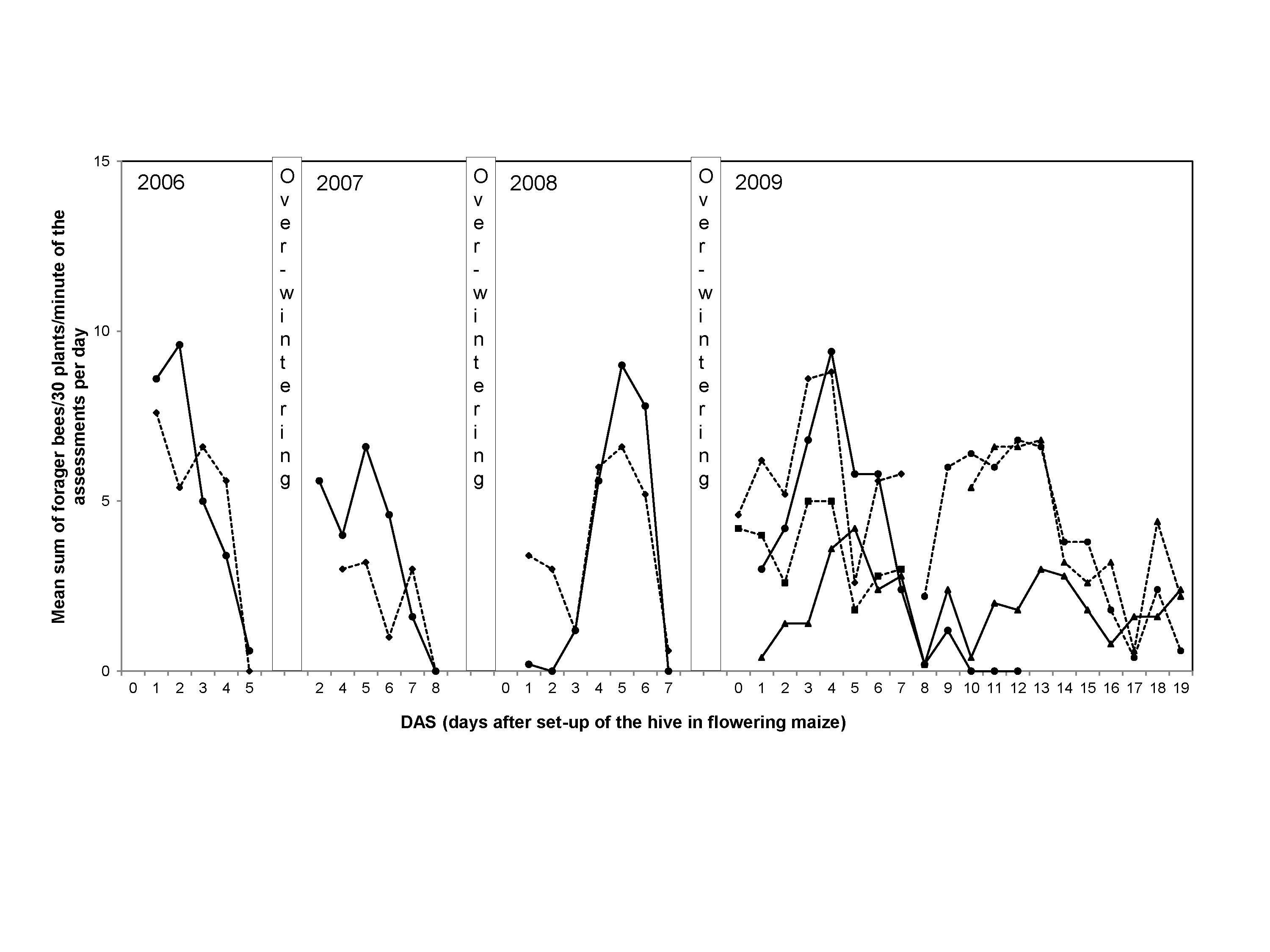

Supplement: Figure S6 — Mean number of forager bees per m2 flowering maize in treated (dashed line) and control (solid line) fields during the time of exposure in the Lorraine region of France from 2006 to 2009. (TIFF) [file pone.0077193.s006.tiff]

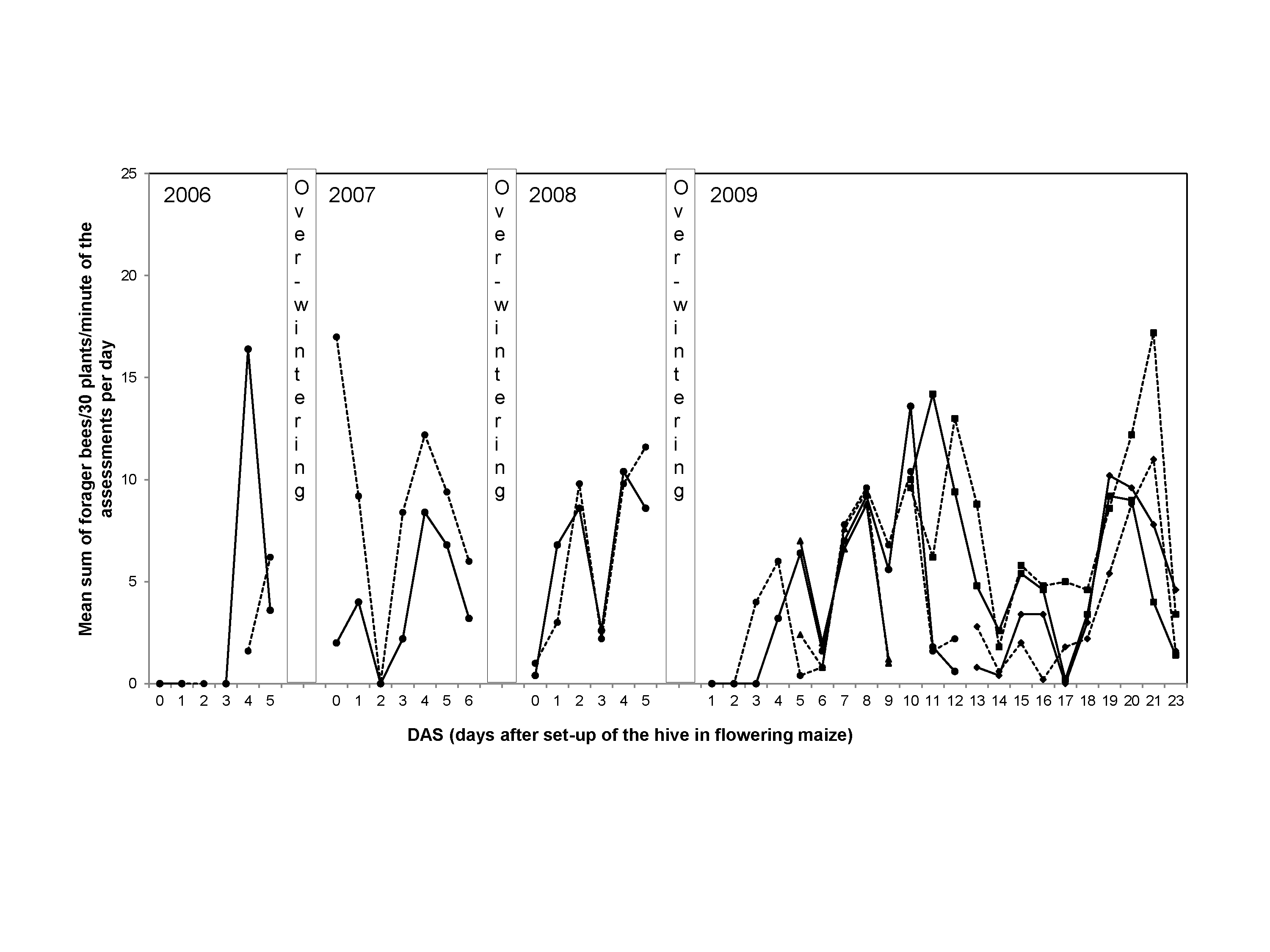

Supplement: Figure S7 — Mean number of forager bees per m2 flowering maize in treated (dashed line) and control (solid line) fields during the time of exposure in the Aveyron region of France from 2006 to 2009. (TIFF) [file pone.0077193.s007.tiff]

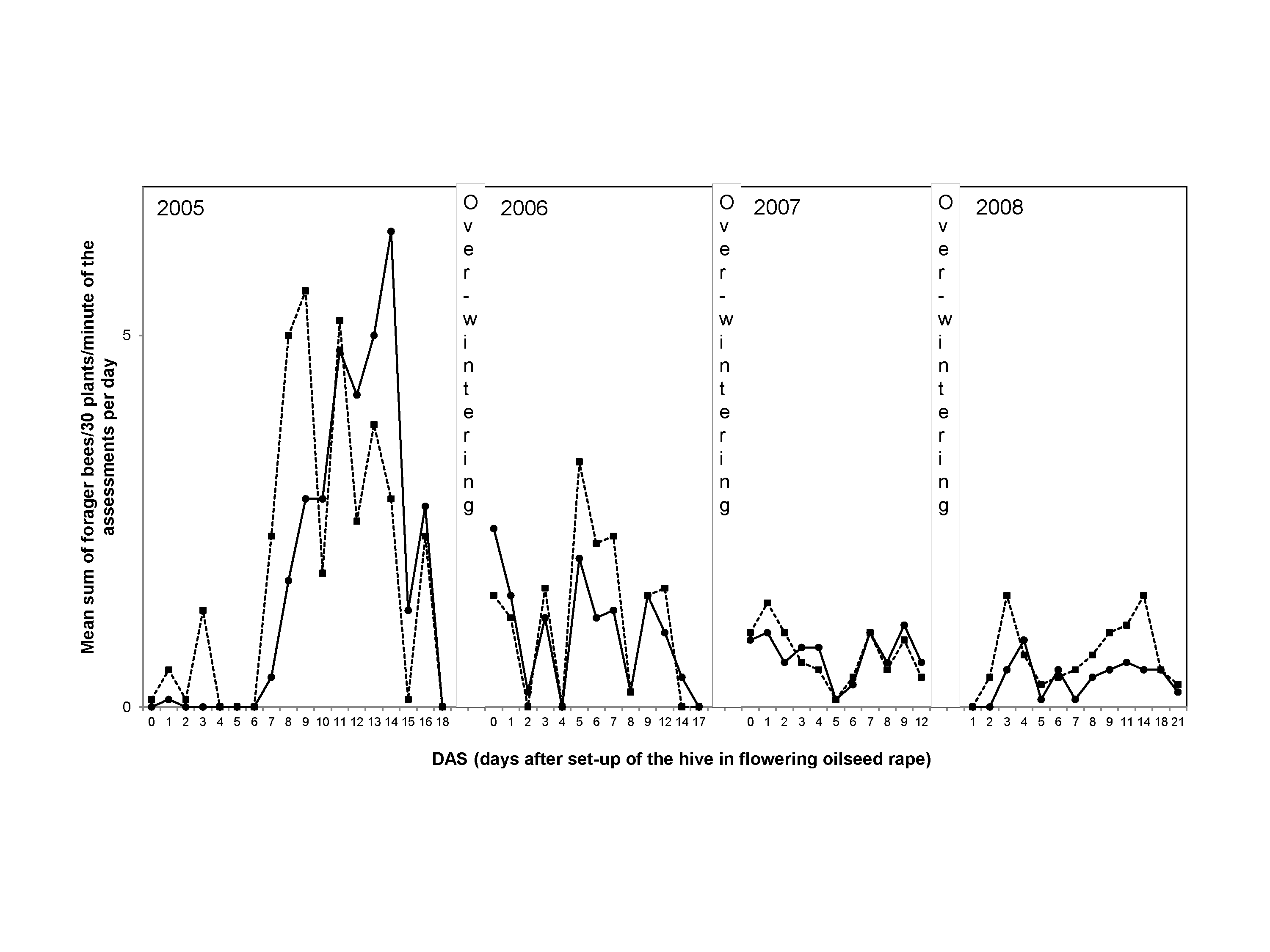

Supplement: Figure S8 — Mean number of forager bees per m2 flowering oilseed rape in treated (dashed line) and control (solid line) fields during the time of exposure in the Picardie region of France from 2005 to 2008. (TIFF) [file pone.0077193.s008.tiff]

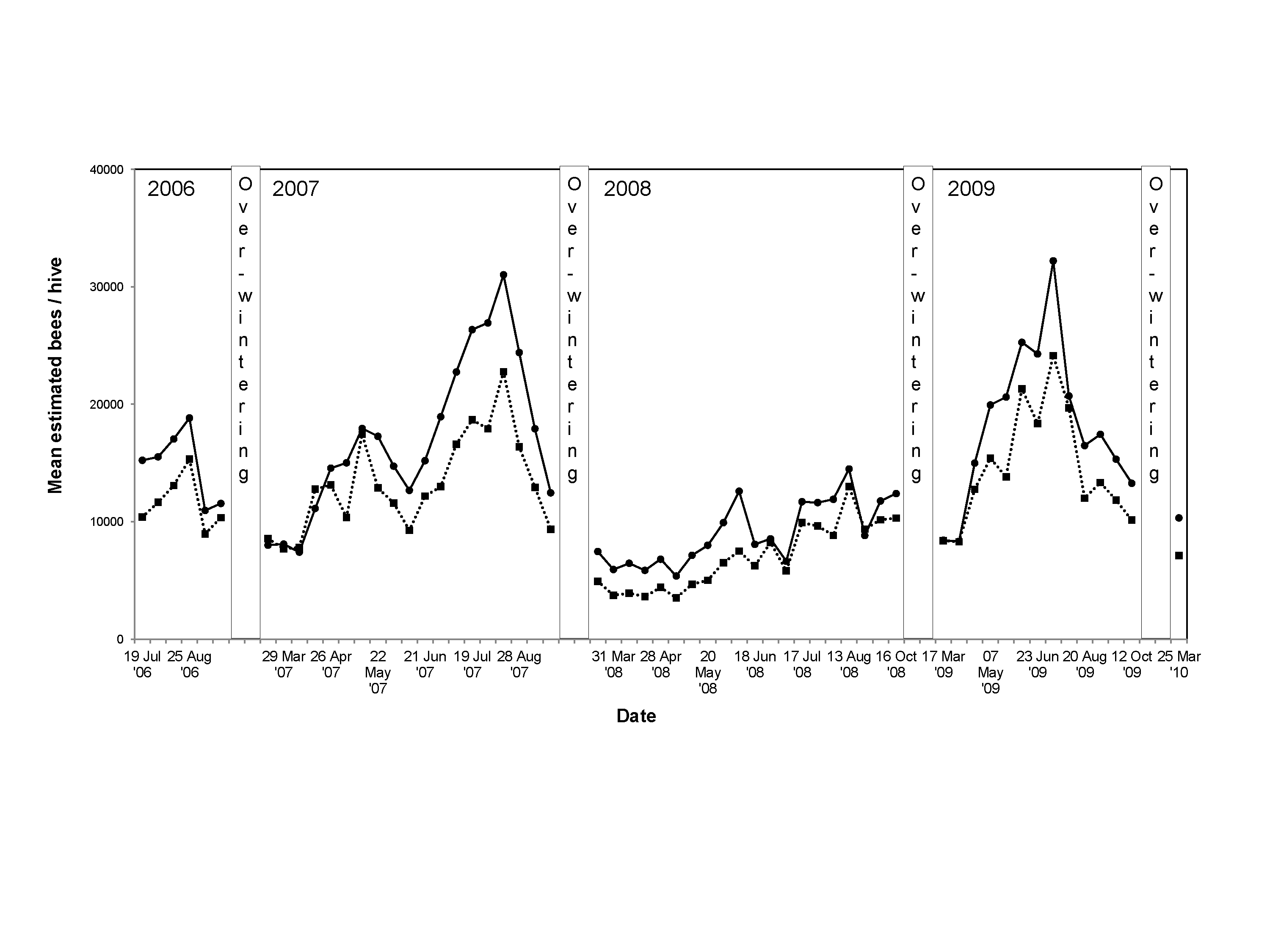

Supplement: Figure S9 — Strength of honey bee colonies exposed to treated (dashed line) and control (solid line) maize fields in the Alsace region of France during the four years of observations including the last overwintering. (TIFF) [file pone.0077193.s009.tiff]

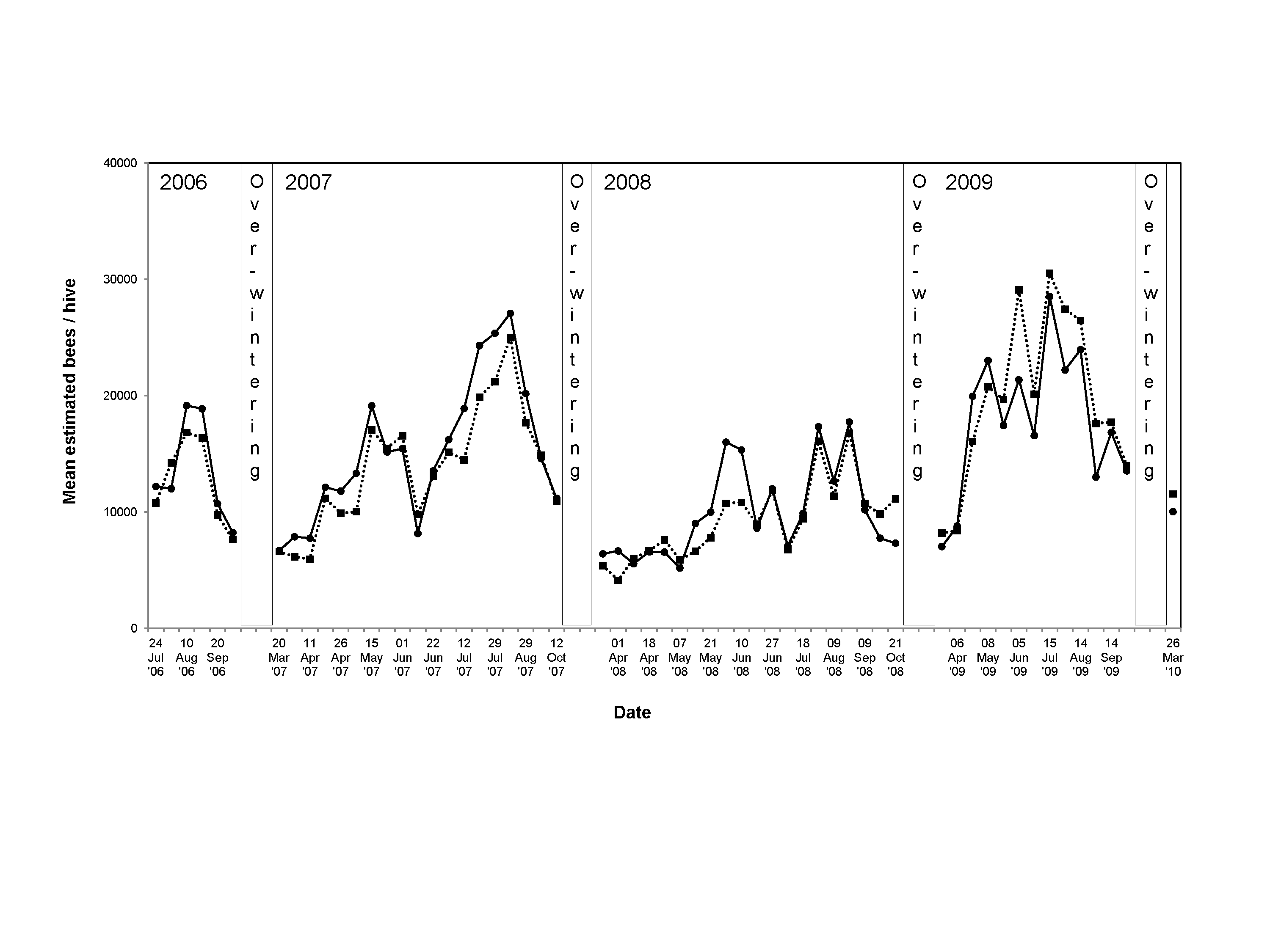

Supplement: Figure S10 — Strength of honey bee colonies exposed to treated (dashed line) and control (solid line) maize fields in the Lorraine region of France during the four years of observations including the last overwintering. (TIFF) [file pone.0077193.s010.tiff]

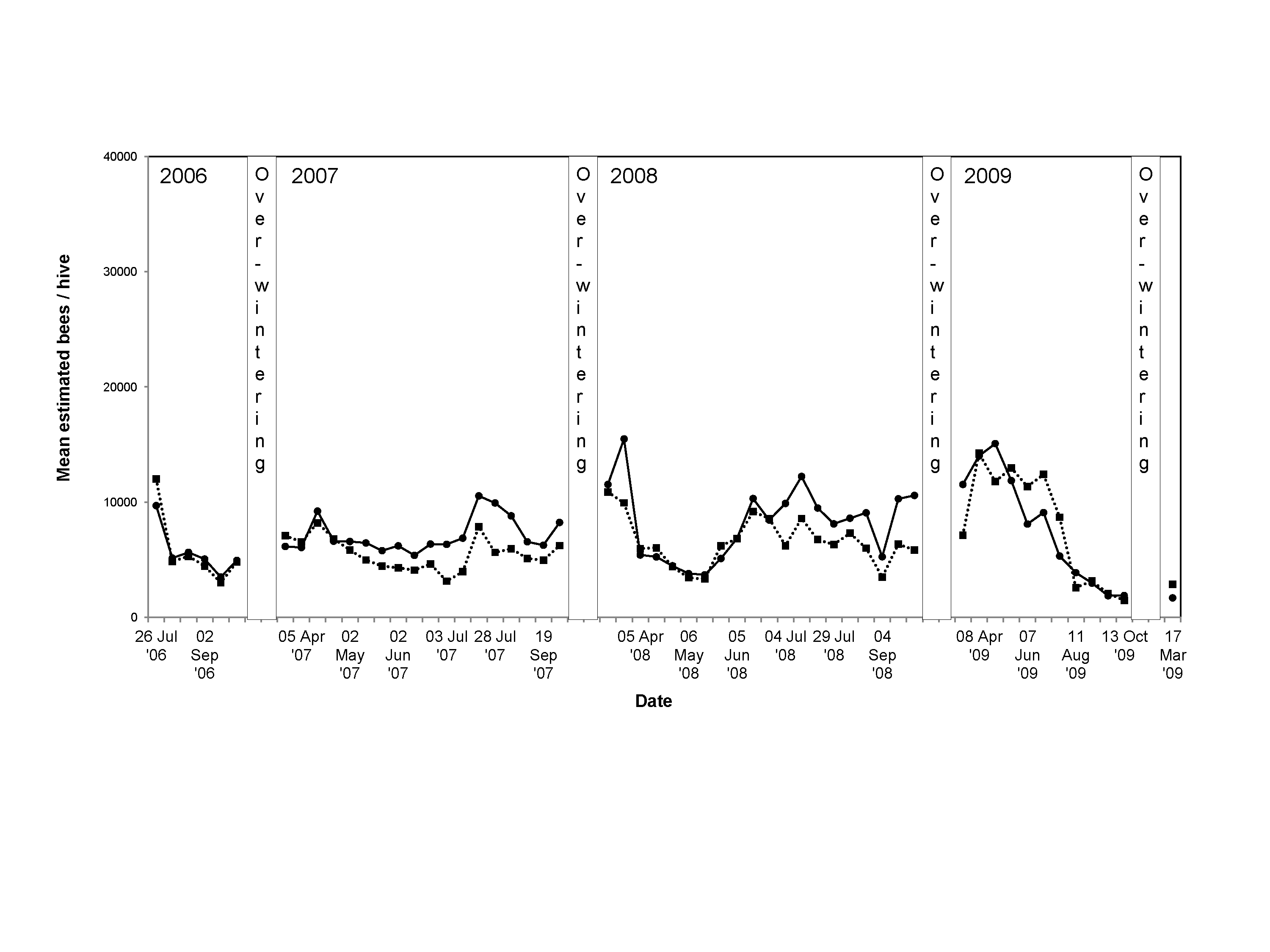

Supplement: Figure S11 — Strength of honey bee colonies exposed to treated (dashed line) and control (solid line) maize fields in the Aveyron region of France during the four years of observations including the last overwintering. (TIFF) [file pone.0077193.s011.tiff]

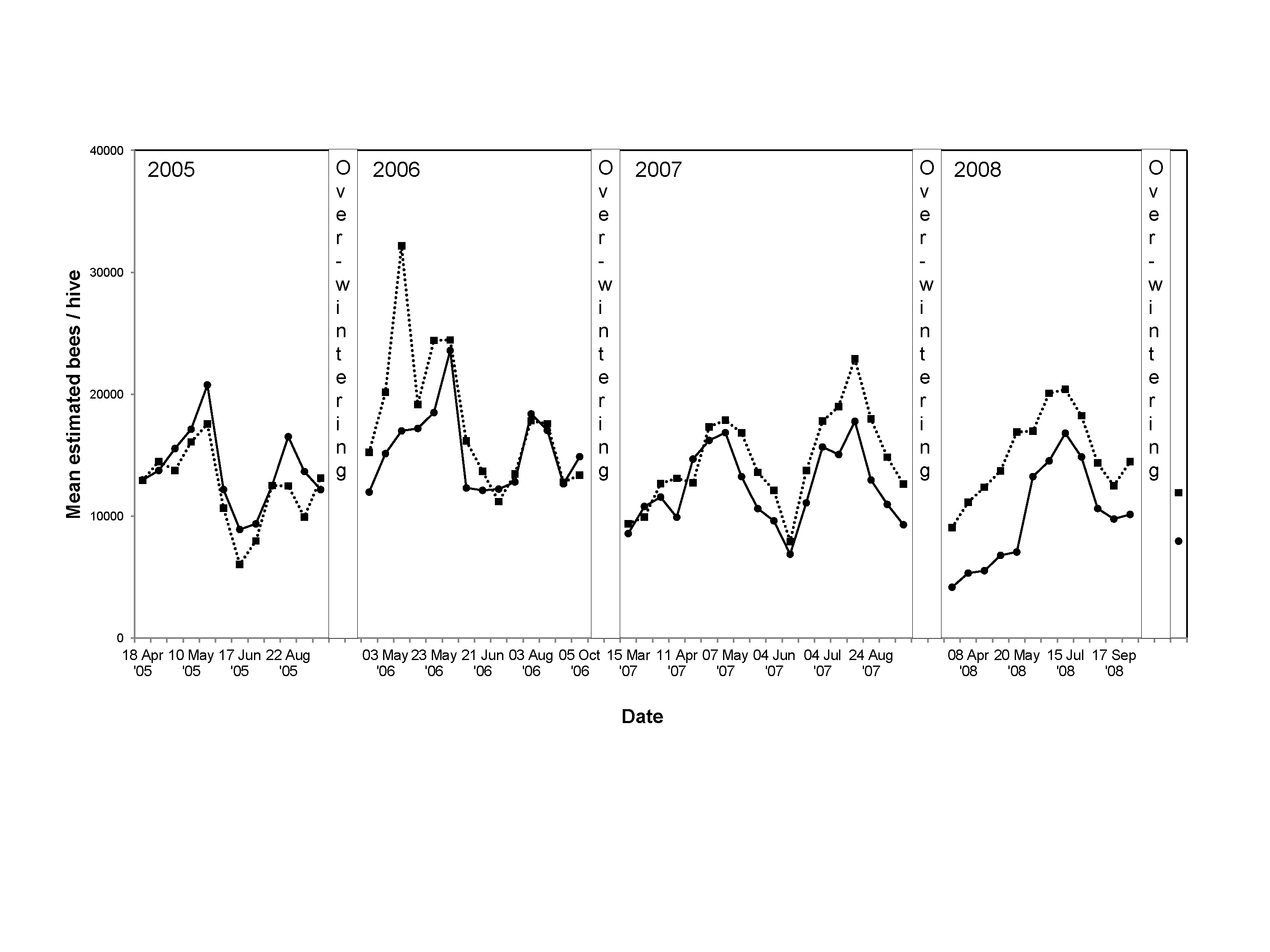

Supplement: Figure S12 — Strength of honey bee colonies exposed to treated (dashed line) and control (solid line) oilseed rape fields in the Picardie region of France during the four years of observations including the last overwintering. (TIFF) [file pone.0077193.s012.tiff]

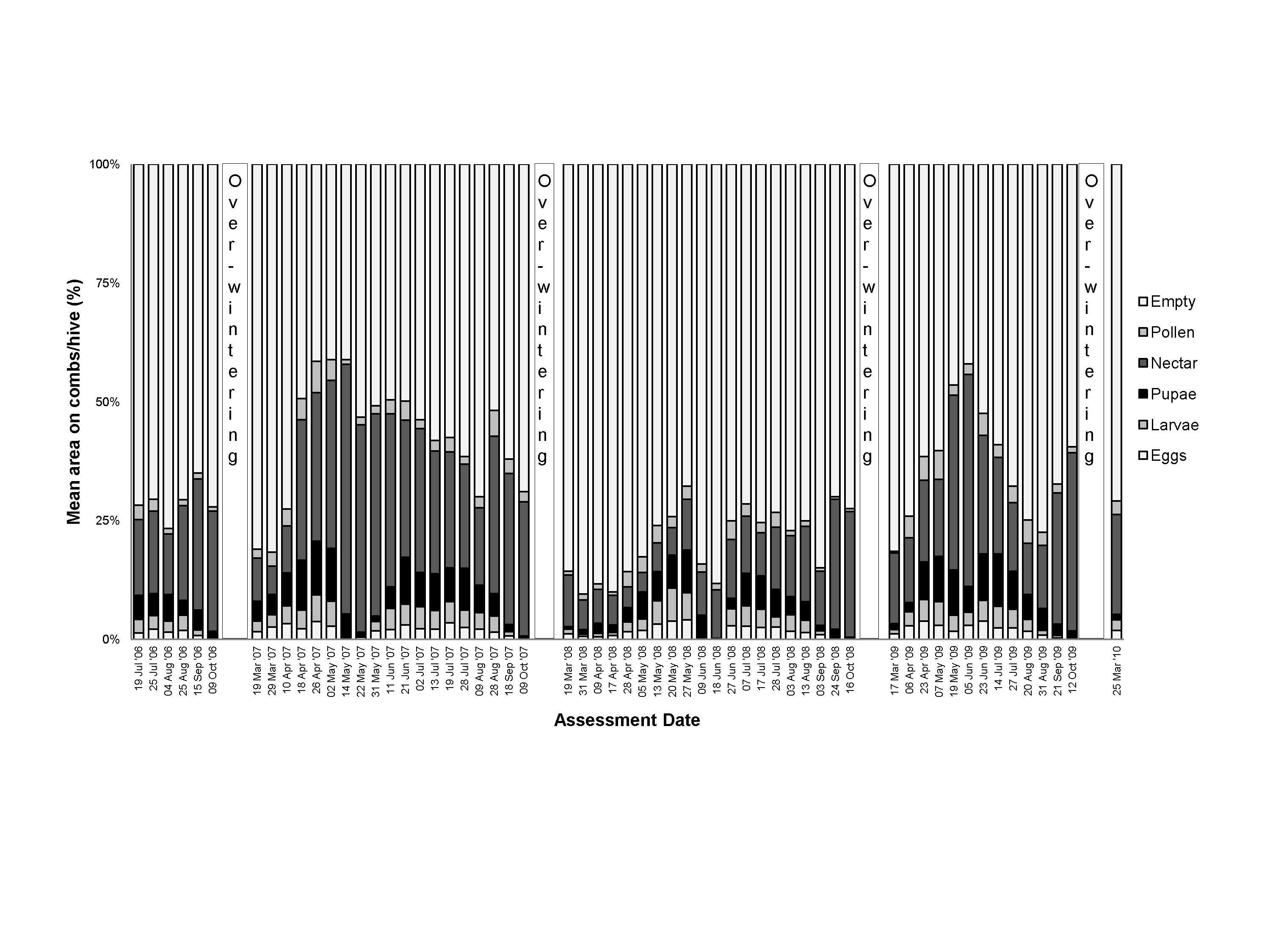

Supplement: Figure S13 — Mean area on combs (%) of brood (eggs, larvae and pupae) and food (nectar and pollen) of 6 colonies exposed to treated maize in the Alsace region of France over 4 years. (TIFF) [file pone.0077193.s013.tiff]

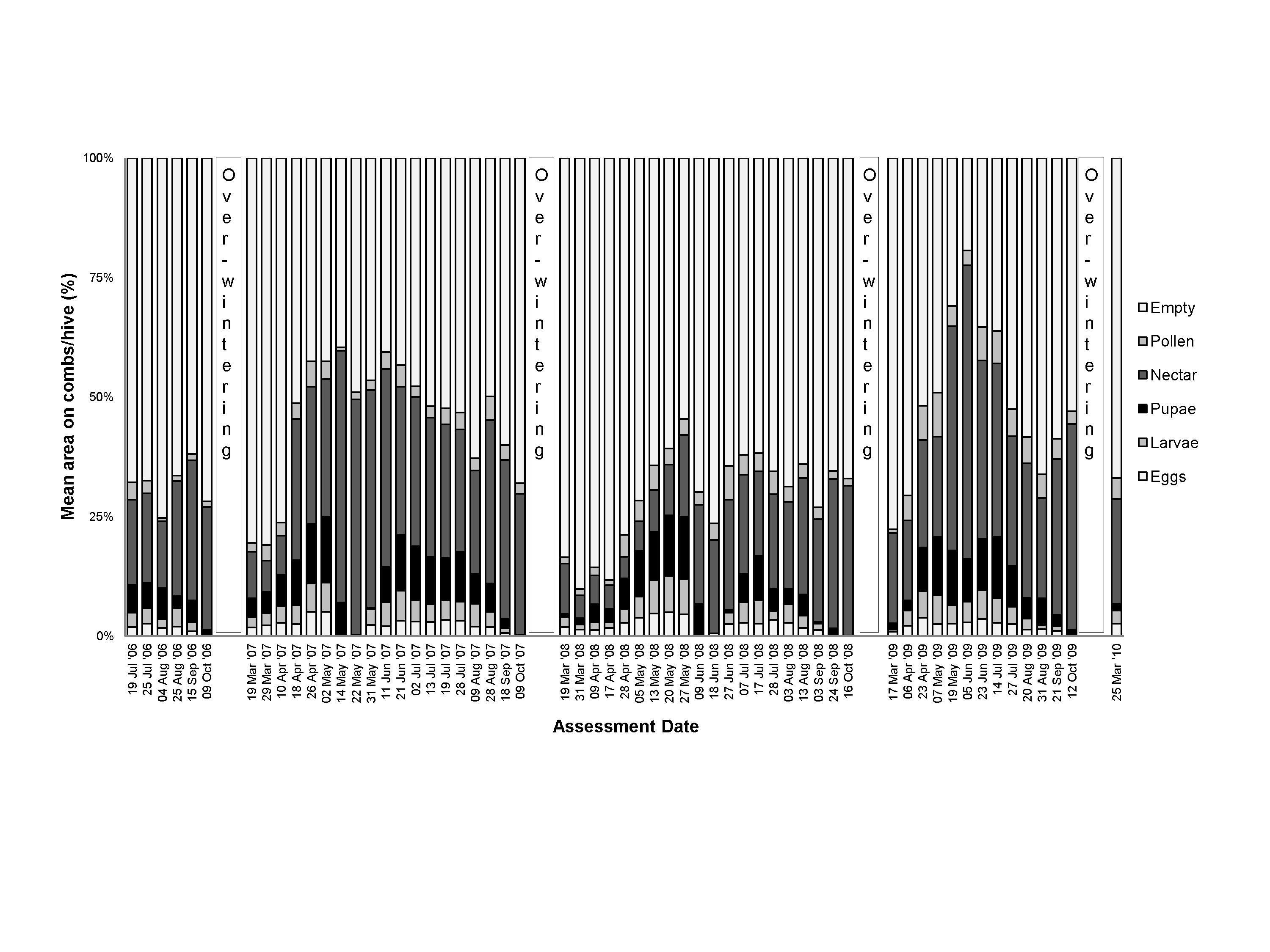

Supplement: Figure S14 — Mean area on combs (%) of brood (eggs, larvae and pupae) and food (nectar and pollen) of 6 colonies exposed to control maize in the Alsace region of France over 4 years. (TIFF) [file pone.0077193.s014.tiff]

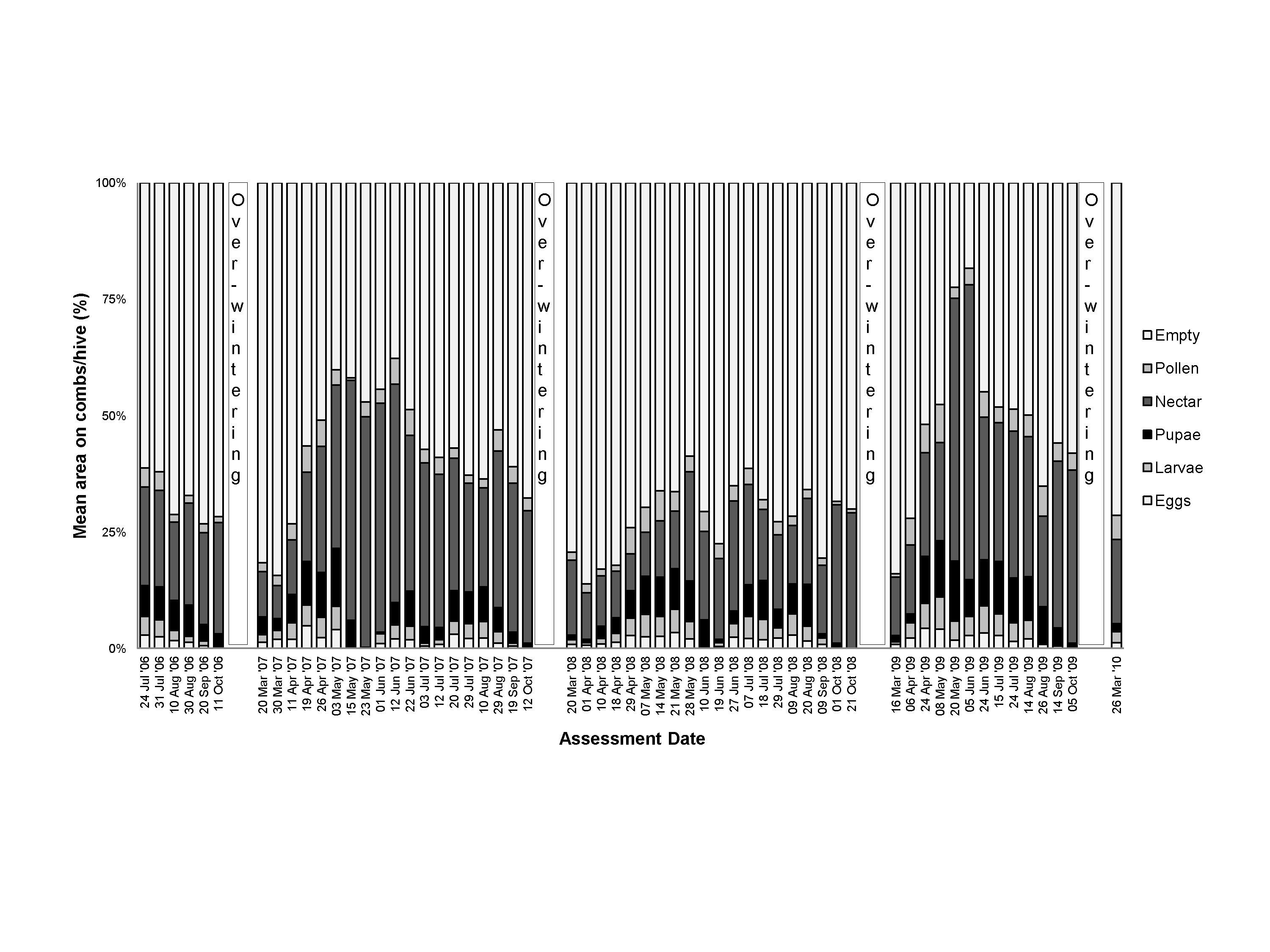

Supplement: Figure S15 — Mean area on combs (%) of brood (eggs, larvae and pupae) and food (nectar and pollen) of 6 colonies exposed to treated maize in the Lorraine region of France over 4 years. (TIFF) [file pone.0077193.s015.tiff]

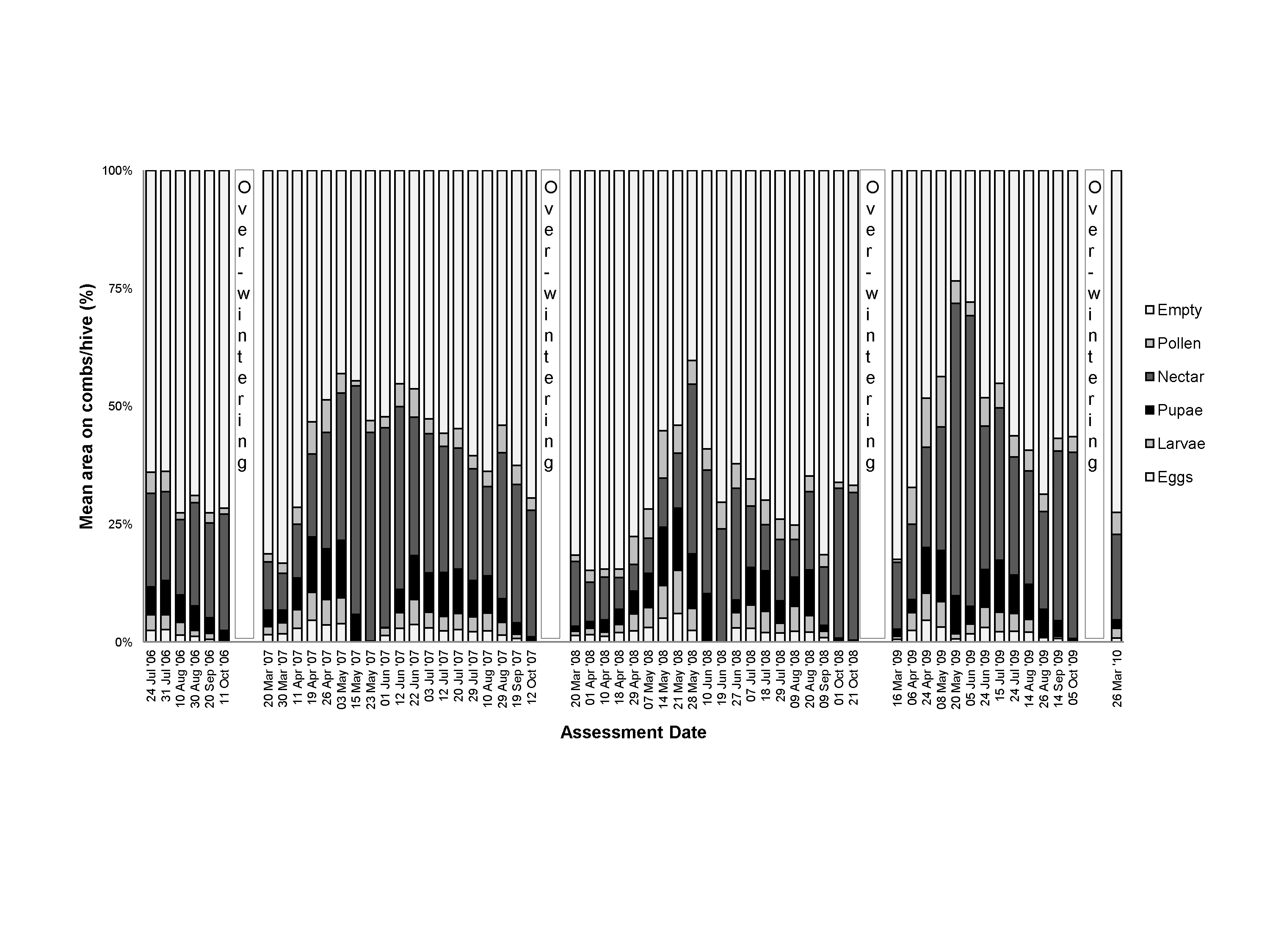

Supplement: Figure S16 — Mean area on combs (%) of brood (eggs, larvae and pupae) and food (nectar and pollen) of 6 colonies exposed to control maize in the Lorraine region of France over 4 years. (TIFF) [file pone.0077193.s016.tiff]

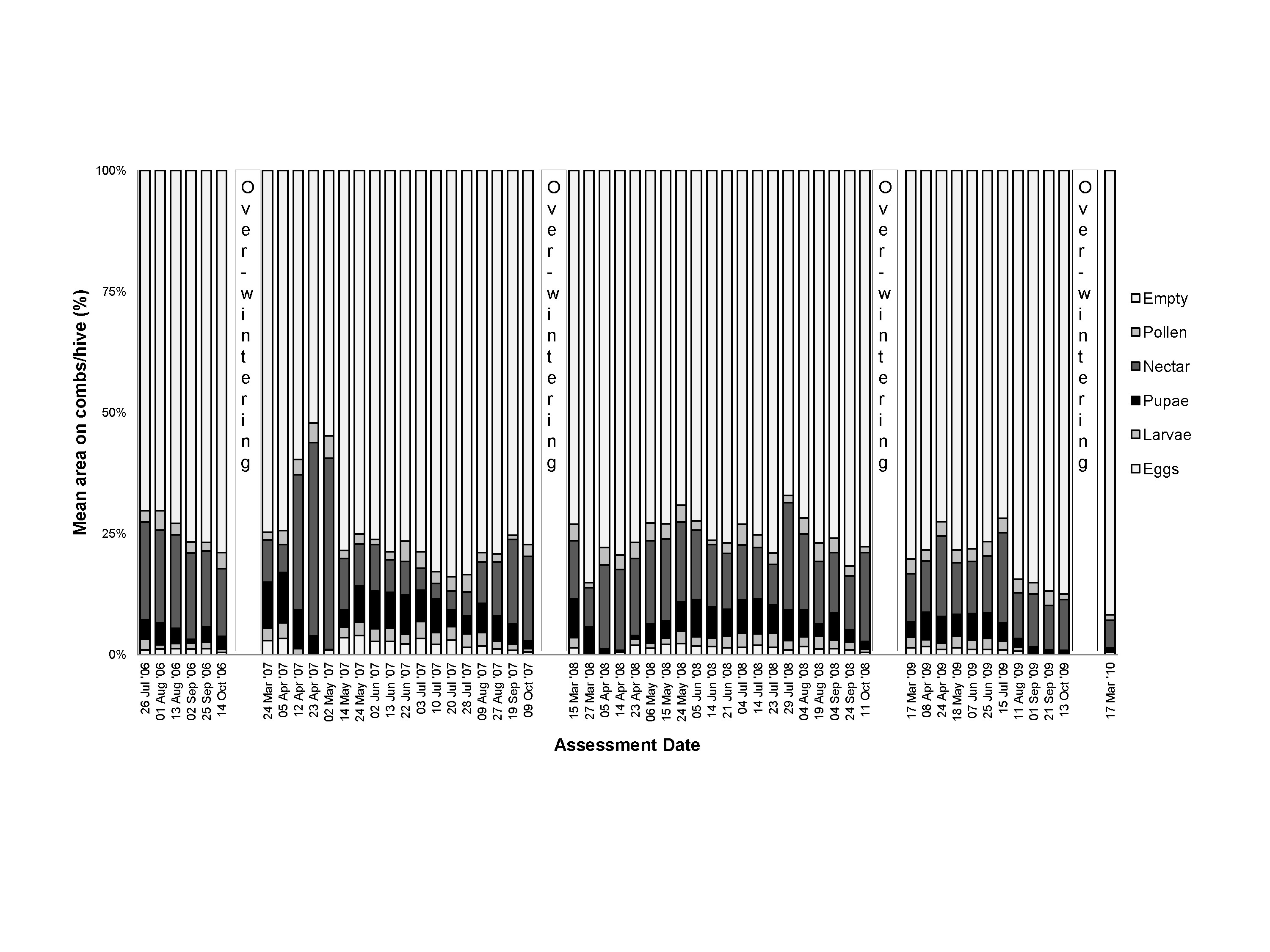

Supplement: Figure S17 — Mean area on combs (%) of brood (eggs, larvae and pupae) and food (nectar and pollen) of 6 colonies exposed to treated maize in the Aveyron region of France over 4 years. (TIFF) [file pone.0077193.s017.tiff]

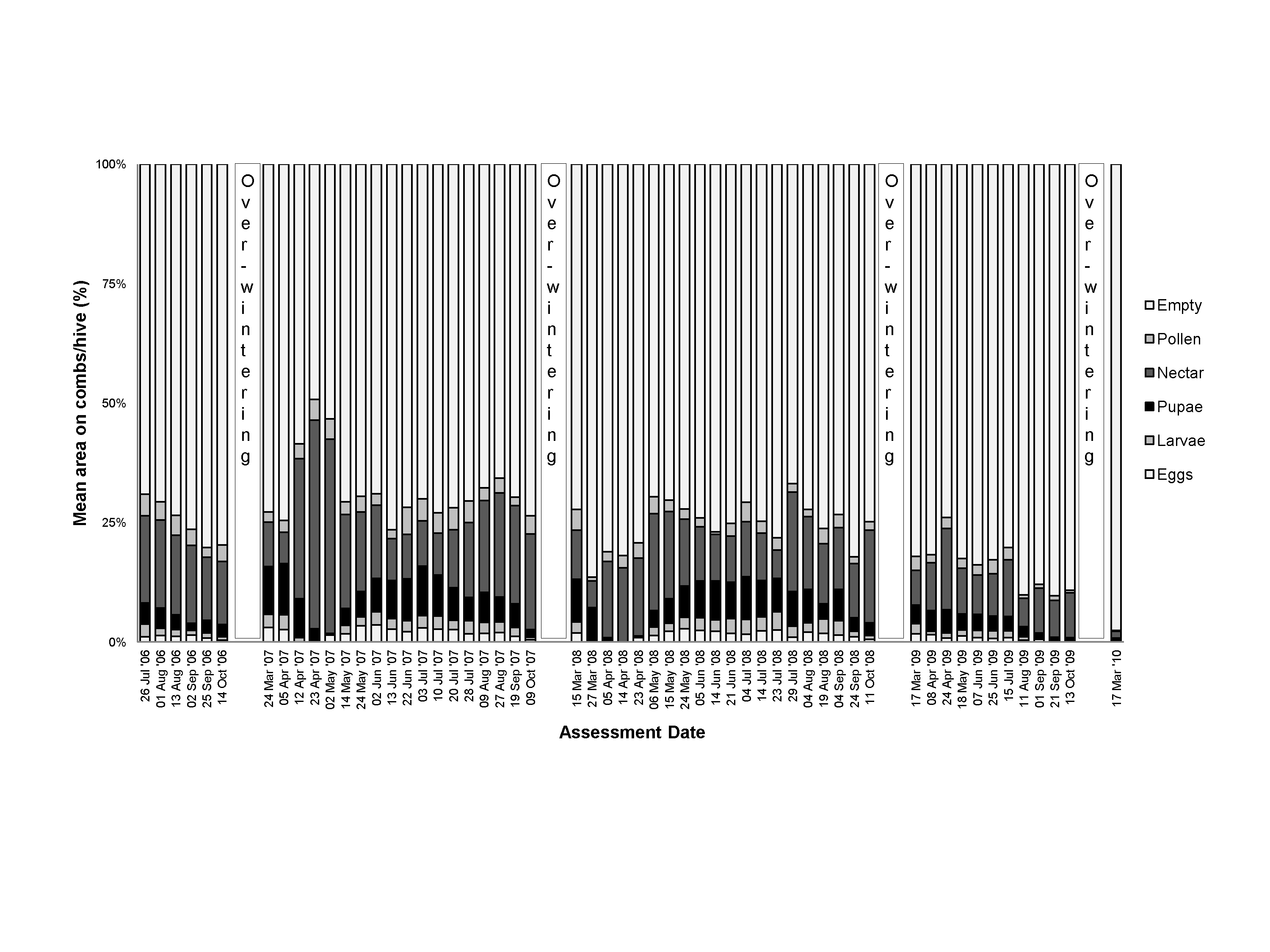

Supplement: Figure S18 — Mean area on combs (%) of brood (eggs, larvae and pupae) and food (nectar and pollen) of 6 colonies exposed to control maize in the Aveyron region of France over 4 years. (TIFF) [file pone.0077193.s018.tiff]

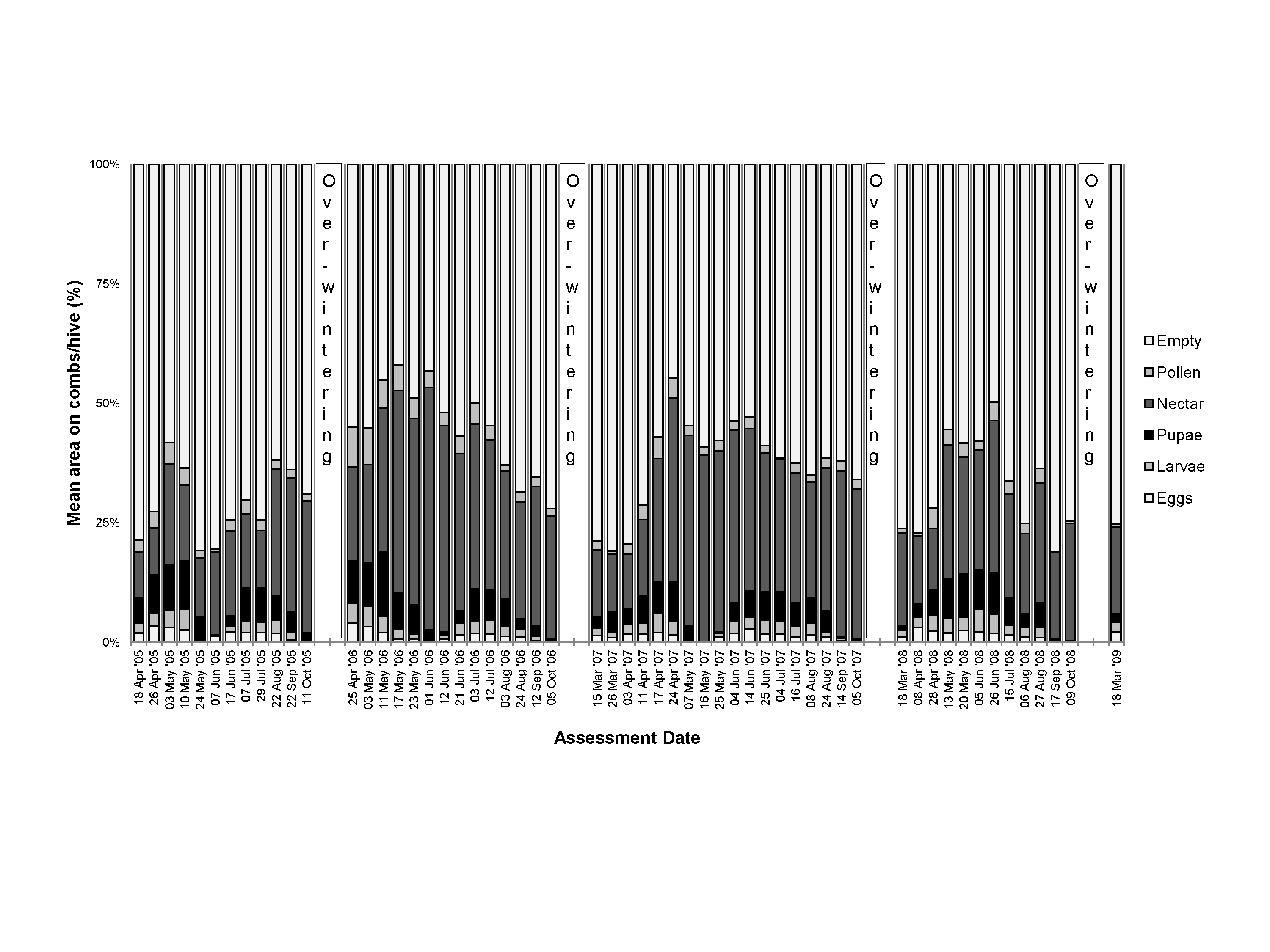

Supplement: Figure S19 — Mean area on combs (%) of brood (eggs, larvae and pupae) and food (nectar and pollen) of 6 colonies exposed to treated oilseed rape in the Picardie region of France over 4 years. (TIFF) [file pone.0077193.s019.tiff]

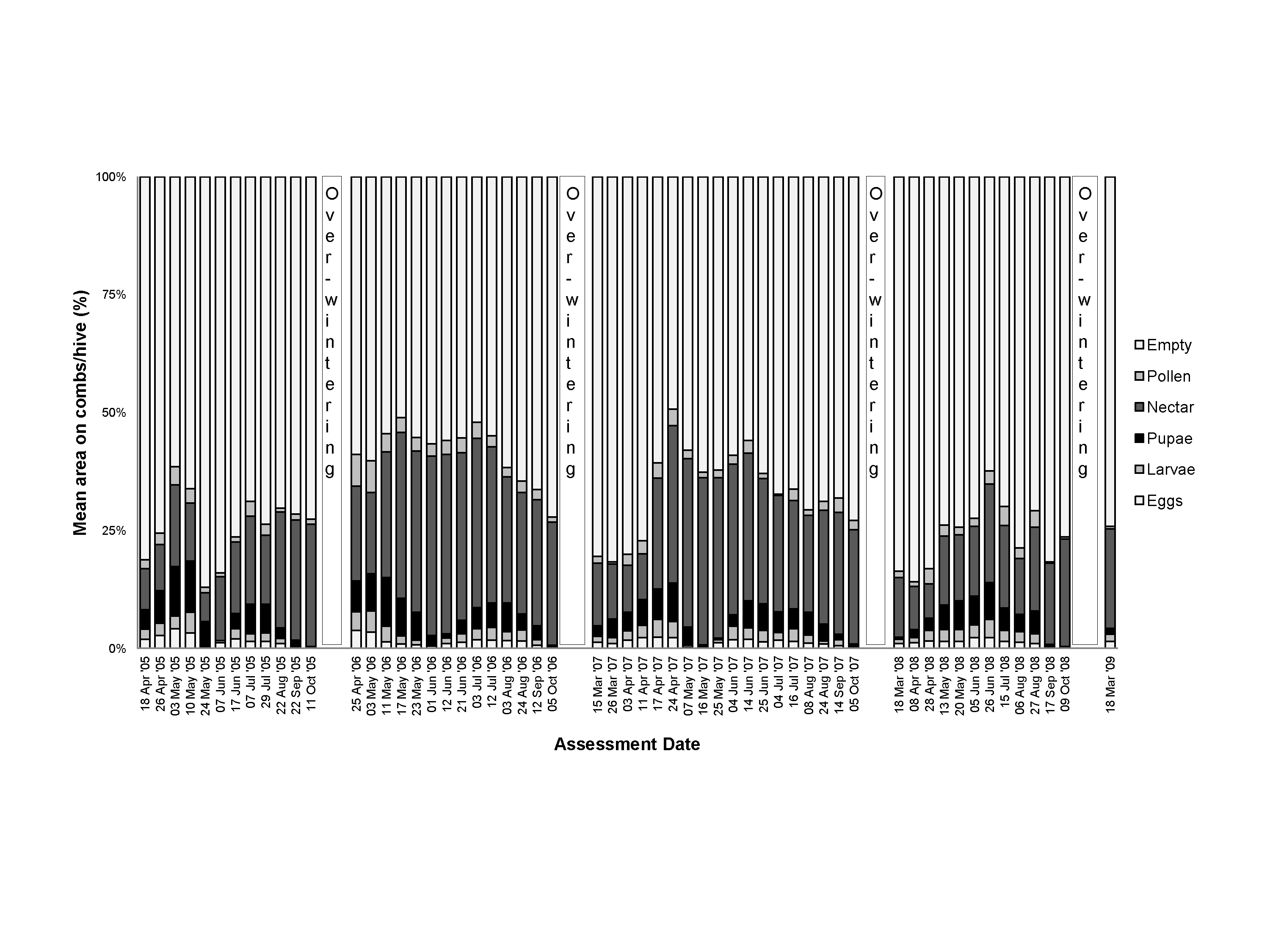

Supplement: Figure S20 — Mean area on combs (%) of brood (eggs, larvae and pupae) and food (nectar and pollen) of 6 colonies exposed to control oilseed rape in the Picardie region of France over 4 years. (TIFF) [file pone.0077193.s020.tiff]

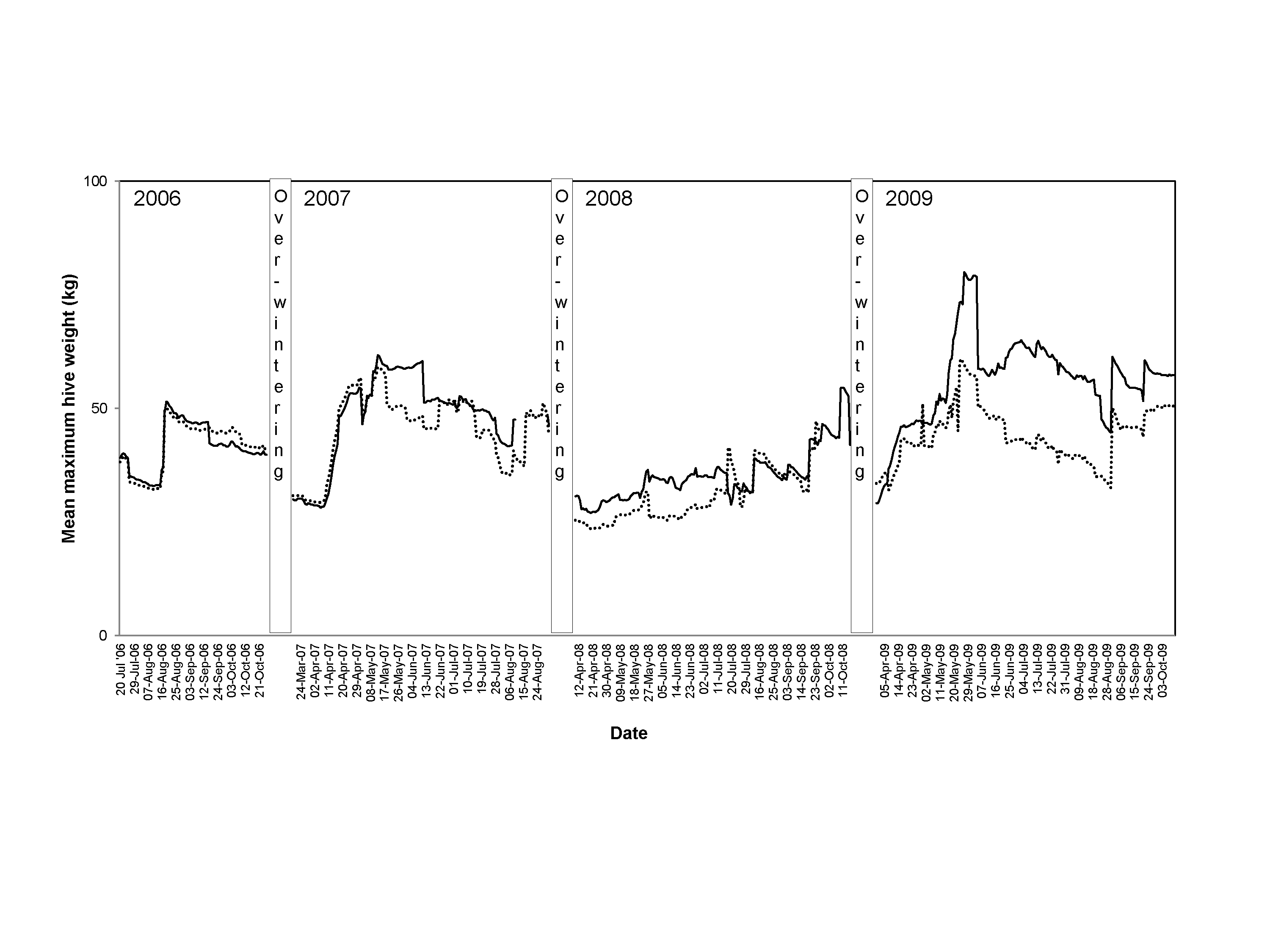

Supplement: Figure S21 — Mean hive weight (kg) during time of assessments of colonies in treated (dashed line) and control (solid line) maize fields in the Alsace region of France. (TIFF) [file pone.0077193.s021.tiff]

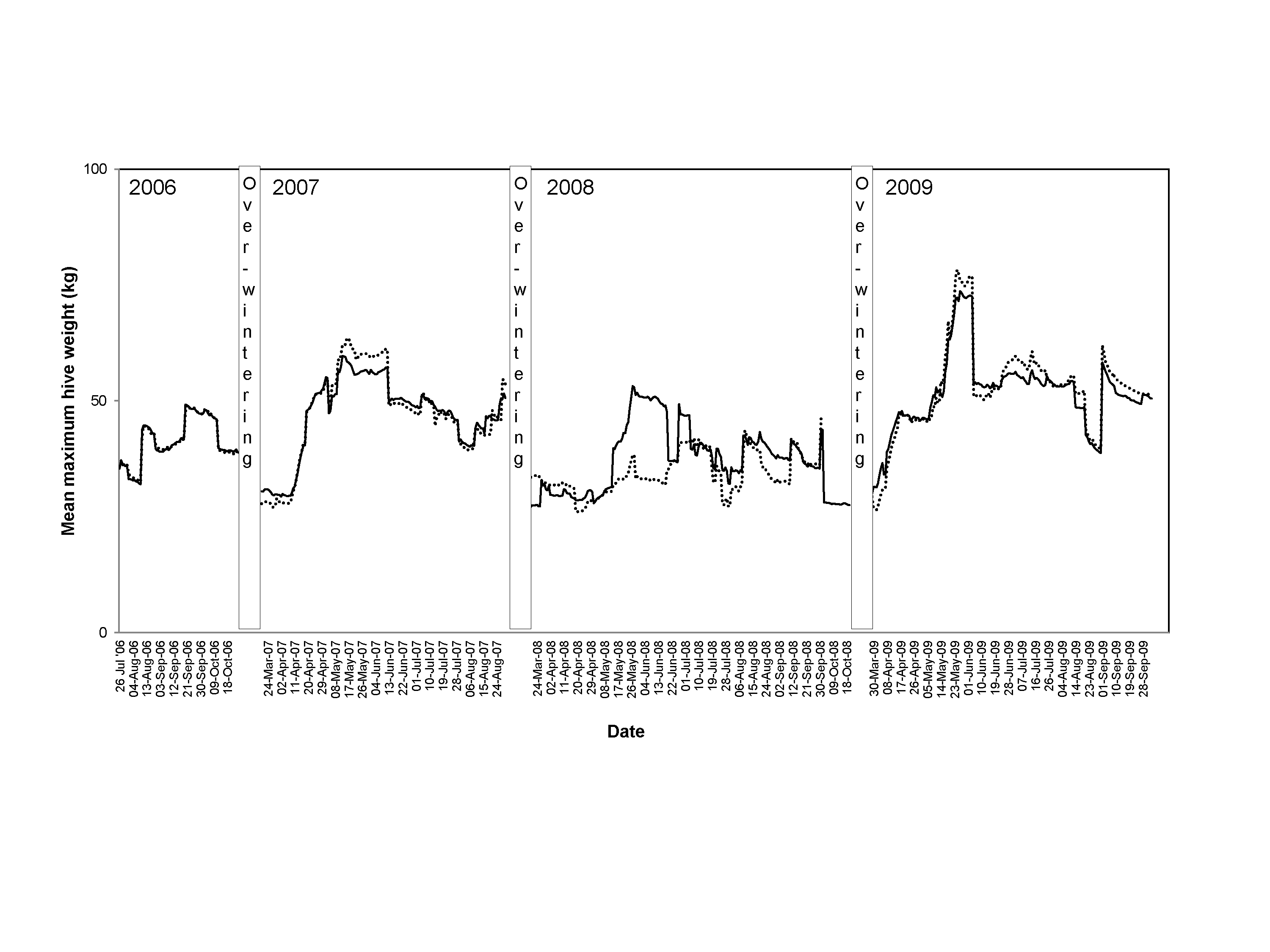

Supplement: Figure S22 — Mean hive weight (kg) during time of assessments of colonies in treated (dashed line) and control (solid line) maize fields in the Lorraine region of France. (TIFF) [file pone.0077193.s022.tiff]

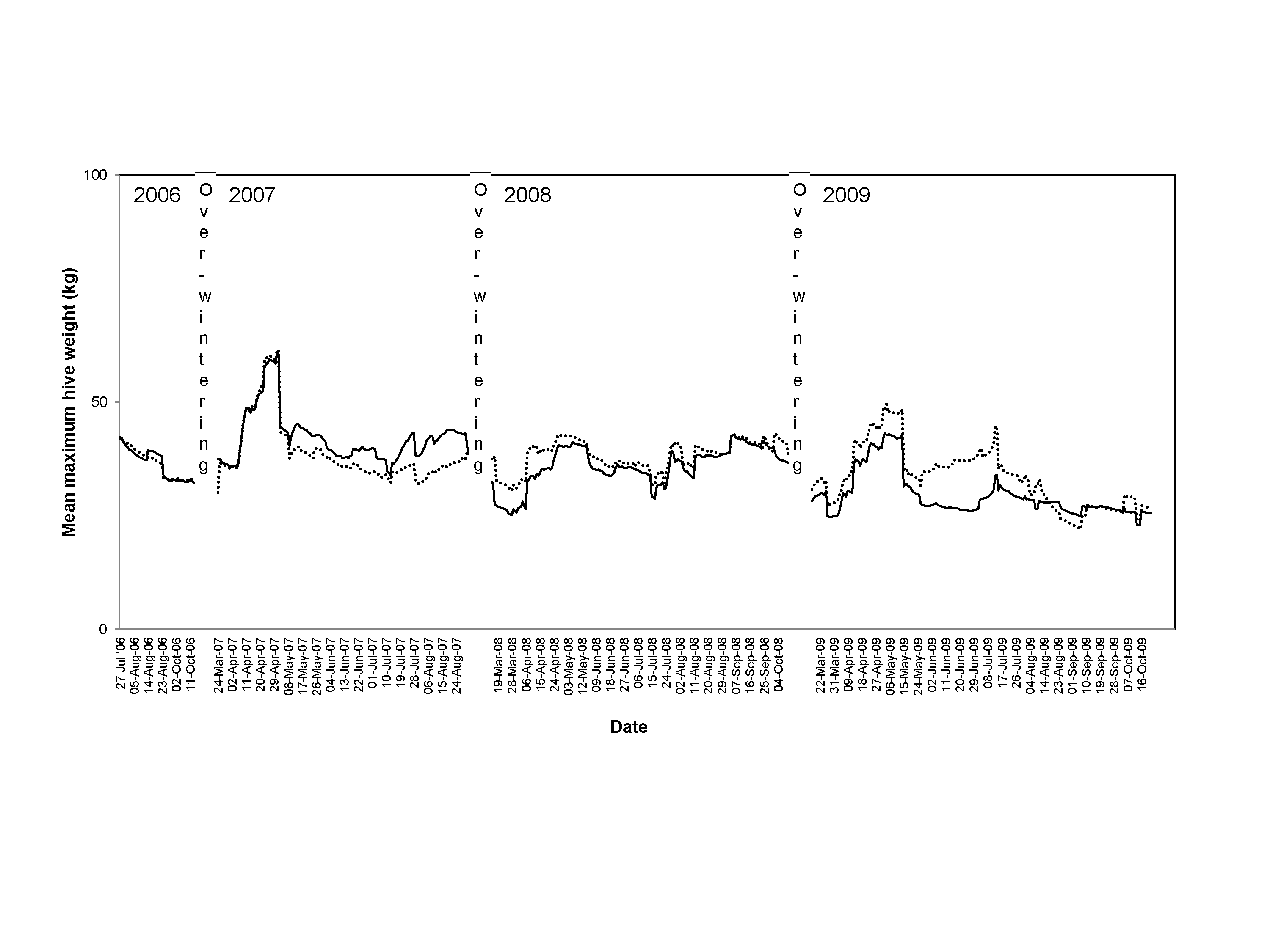

Supplement: Figure S23 — Mean hive weight (kg) during time of assessments of colonies in treated (dashed line) and control (solid line) maize fields in the Aveyron region of France. (TIFF) [file pone.0077193.s023.tiff]

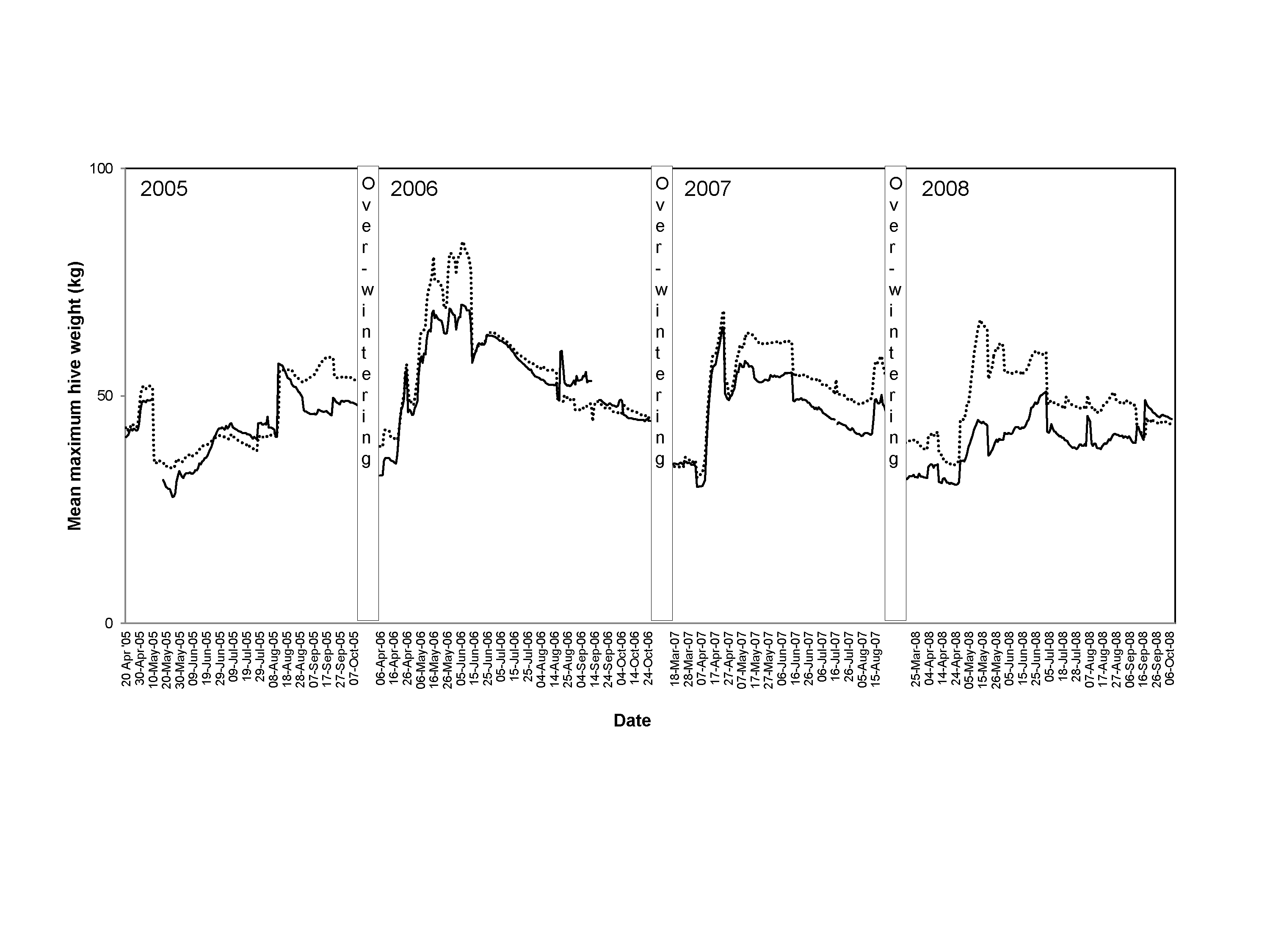

Supplement: Figure S24 — Mean hive weight (kg) during time of assessments of colonies in treated (dashed line) and control (solid line) oilseed rape fields in the Picardie region of France. (TIFF) [file pone.0077193.s024.tiff]
