# Supplementary material for: A Four-Year Field Program Investigating Long-Term Effects of Repeated Exposure of Honey Bee Colonies to Flowering Crops Treated with Thiamethoxam
Source: PLoS One. 2013 Oct 23;8(10):e77193. doi: 10.1371/journal.pone.0077193 (PMC3806756; doi:10.1371/journal.pone.0077193)
Supplement: Table S1 — Median thiamethoxam and CGA322704 residue values (and range in parentheses) found in plants, bee and hive pollen following exposure in maize in tunnels, and bee pollen and nectar and hive pollen and nectar following exposure in oilseed rape in tunnels. (DOCX) [file pone.0077193.s025.docx]

**Full Title: A four year field program investigating long term effects of repeated exposure of honey bee colonies to flowering crops treated with thiamethoxam**

Supporting Information

**Table S1.** Median thiamethoxam and CGA322704 residue values (and range in parentheses) found in plants, bee and hive pollen following exposure in maize in tunnels, and bee pollen and nectar and hive pollen and nectar following exposure in oilseed rape in tunnels.

| **Crop** | **Location** | **Sample type** | **Median Thiamethoxam residue^1^ (µg/kg)**  LOQ = 1 µg/kg | | **Median CGA322704 residue^1^ (µg/kg)** LOQ = 1 µg kg | |
| --- | --- | --- | --- | --- | --- | --- |
|  |  |  | **Control** | **Treated** | **Control** | **Treated** |
| Maize  (first year) | Alsace | Plant | <1 | 13 (10-18) | <1 | 12 (10-16) |
|  |  | Pollen (bee) | <1 | 7 (5-12) | <1 | 4 (3-7) |
|  |  | Pollen (hive) | <1 | <1 (<1-4) | <1 | <1 (<1-6) |
| Maize  (first year) | Champagne | Plant | <1 | 4 (3-6) | <1 | 3 (2-5) |
|  |  | Pollen (bee) | <1 | 3 (1-4) | <1 | 2 (2-3) |
|  |  | Pollen (hive) | <1 | <1 (<1-2) | <1 | <1 (<1-3) |
| Maize  (first year) | Midi-Pyrénées | Plant | <1 | 14 (9-20) | <1 | 6 (4-8) |
|  |  | Pollen (bee) | <1 | 5  (1-12) | <1 | 1 (<1 – 2) |
|  |  | Pollen (hive) | <1 | <1 (<1-2) | <1 | <1 (<1-1) |
| Maize (second year) | Alsace | Plant | <1 | 10 (3-12) | <1 | 7 (2-8) |
|  |  | Pollen (bee) | <1 | 1 (<1-2) | <1 | 1 (<1-2) |
|  |  | Pollen (hive) | <1 | <1 | <1 | <1 |
| Maize (second year) | Champagne | Plant | <1 | 2 (2-4) | <1 | 2 (2-4) |
|  |  | Pollen (bee) | <1 | 2 (<1-2) | <1 | 1  (<1-2) |
|  |  | Pollen (hive) | <1 | <1 (<1-1) | <1 | <1 (<1-1) |
| Maize (second year) | Midi-Pyrénées | Plant | <1 | 26 (17-50) | <1 | 8 (6-12) |
|  |  | Pollen (bee) | <1 | 3 (1-8) | <1 | 2 (1-3) |
|  |  | Pollen (hive) | <1 | <1 (<1-1) | <1 | <1 |
| Oilseed rape | Alsace | Plants | <1 | 3  (<1-7) | <1 | 1  (<1-2) |
|  |  | Pollen (bee) | <1 | 3 (2-4) | <1 | <1 |
|  |  | Nectar (bee) | <0.5^2^ | 2^2^  (2-4) | <1 | <1 |
|  |  | Pollen (hive) | <1 | <1  (<1-3) | <1 | <1 |
|  |  | Nectar (hive) | <0.5^2^ | <0.5^2^ | <1 | <1 |
| Oilseed rape | Picardie | Plants | <1 | <1 | <1 | <1 |
|  |  | Pollen (bee) | <1 | <1  (<1-1) | <1 | <1 |
|  |  | Nectar (bee) | <0.5^2^ | 0.65^2^  (0.5-1.4) | <1 | <1 |
|  |  | Pollen (hive) | <1 | <1 | <1 | <1 |
|  |  | Nectar (hive) | <0.5^2^ | <0.5^2^ | <1 | <1 |
| Oilseed rape | Midi-Pyrénées | Plants | <1 | 2  (1-5) | <1 | <1  (<1-1) |
|  |  | Pollen (bee) | <1 | 2  (<1-4) | <1 | <1 |
|  |  | Nectar (bee) | <0.5^2^ | 2^2^  (1-4) | <1 | <1  (<1-1) |
|  |  | Pollen (hive) | <1 | <1  (<1-2) | <1 | <1 |
|  |  | Nectar (hive) | <0.5^2^ | <0.5^2^  (<0.5-0.9) | <1 | <1 |
| Oilseed rape following barley | Picardie | Plants | <1 | <1 | <1 | <1  (<1-1) |
|  |  | Pollen (bee) | <1 | <1 | <1 | <1 |
|  |  | Nectar (bee) | <0.5^2^ | 1.4^2^  (<0.5-2.2) | <1 | <1 |
|  |  | Pollen (hive) | <1 | <1  (<1-1) | <1 | <1 (<1-4) |
|  |  | Nectar (hive) | <0.5^2^ | <0.5^2^ | <1 | <1 |
| Oilseed rape  following barley | Champagne | Plants | <1 | <1  (<1-2) | <1 | <1 (<1-1) |
|  |  | Pollen (bee) | <1 | 1  (<1-3) | <1 | <1 |
|  |  | Nectar (bee) | <0.5^2^ | 0.7^2^ (<0.5-2.4) | <1 | <1 (<1-1) |
|  |  | Pollen (hive) | <1 | <1 (<1-1) | <1 | <1 |
|  |  | Nectar (hive) | <0.5^2^ | <0.5^2^ | <1 | <1 |
| Oilseed rape  Following barley | Midi-Pyrénées | Plants | <1 | 3  <1-5) | <1 | 2  (1-3) |
|  |  | Pollen (bee) | <1 | 3.5 (1-6) | <1 | <1  (<1-2) |
|  |  | Nectar (bee) | <0.5^2^ | 2.4 (0.9-4.6) | <1 | <1 |
|  |  | Pollen (hive) | <1 | <1  (<1-3) | <1 | <1  (<1-1) |
|  |  | Nectar (hive) | <0.5^2^ | <0.5^2^ (<0.5-2.5) | <1 | <1 |

^1^Range of residue values given in parentheses

^2^LOQ = 0.5 µg/kg
